# Efficacy and safety of Resmetirom, a selective thyroid hormone receptor-β agonist, in the treatment of metabolic dysfunction-associated steatotic liver disease (MASLD): a systematic review and meta-analysis

**DOI:** 10.1038/s41598-024-70242-8

**Published:** 2024-08-26

**Authors:** Renuka Suvarna, Sahana Shetty, Joseph M. Pappachan

**Affiliations:** 1 Department of Endocrinology, Kasturba Medical College, Manipal, Manipal Academy of Higher Education, Manipal, Karnataka 576104 India; 2 Department of Endocrinology and Metabolism, Lancashire Teaching Hospitals NHS Trust, Preston, PR2 9HT United Kingdom

**Keywords:** Resmetirom, Metabolic dysfunction-associated steatotic liver disease, Non-alcoholic fatty liver disease, Non-alcoholic steatohepatitis, Thyroid hormone receptor-β agonist, Endocrinology, Health care

## Abstract

Metabolic dysfunction-associated steatotic liver disease (MASLD) is an important public health problem owing to its high prevalence and associated morbidity and mortality secondary to progressive liver disease and cardiovascular events. Resmetirom, a selective thyroid hormone receptor-β agonist has been developed as a therapeutic modality for MASLD. This systematic review and meta-analysis aimed to evaluate the effectiveness and safety of resmetirom compared to a placebo in the treatment of MASLD. Eligible studies were systematically identified by screening PubMed, Scopus, Web of Science, Cochrane library, Embase, and ClinicalTrials.gov from 2014 to 2024. Only randomized controlled trials comparing the efficacy and safety of resmetirom in the treatment of MASLD against placebo were included in the analysis. Meta-analysis was performed using RevMan 5.4 software. Four studies with low risk of bias and involving a total of 2359 participants were identified. The metanalysis included only three clinical trials with 2234 participants. A significant reduction in MRI-proton density fat fraction (MRI-PDFF) with 80 mg Resmetirom compared to that with placebo [SMD − 27.74 (95% CI − 32.05 to − 32.42), p < 0.00001] at 36–52 weeks as well as at 12–16 weeks [SMD − 30.92 (95% CI − 36.44 to − 25.40), p < 0.00001]. With Resmetirom 100 mg dose at 36–52 weeks [SMD − 36.05 (95% CI − 40.67 to − 31.43), p < 0.00001] and 12–16 weeks [SMD − 36.89 (95% CI − 40.73 to − 33.05), p < 0.00001] were observed. Resmetirom treatment was associated with a significant reduction in LDL-c triglyceride, lipoproteins. and liver enzymes. There was significant reduction FT4 and increase in SHBG and sex steroids with Resmetirom compared to placebo. There was no major difference in the overall treatment emergent adverse events at 80 mg [OR 1.55 (95% CI 0.84 to 2.87), and 100 mg [OR 1.13 (95% CI 0.78 to 1.63), doses of Resmetirom compared to placebo. However, gastrointestinal adverse events diarrhoea and nausea occurred in ≥ 10% in the Resmetirom group compared to placebo at < 12 week. Resmetirom treatment showed modest efficacy in treating MASLD with reduction in MRI-PDFF, LDL-c, triglyceride, lipoproteins, liver enzymes and NASH biomarkers without significant safety concerns. Larger and long-term RCTs may further confirm this promising outcomes of Resmetirom use in MASLD.

## Introduction

Metabolic Dysfunction-associated Steatotic Liver Disease (MASLD) encompasses a spectrum of liver conditions ranging from non-alcoholic fatty liver disease (NAFLD), non-alcoholic steatohepatitis (NASH), fibrosis, cirrhosis, and hepatocellular carcinoma (HCC) in the presence of metabolic syndrome^1^. With the obesity prevalence assuming pandemic proportions, MASLD has become the leading cause of chronic liver disease worldwide, with prevalence estimates varying based on population demographics, diagnostic methods, and risk factors. It affects approximately one third of the global population. In Western countries, MASLD prevalence ranges from 20 to 30%, while in Asia, it varies from 15 to 45%^2^. The prevalence of MASLD is closely associated with the rising rates of obesity, type 2 diabetes, metabolic syndrome, and sedentary lifestyles^3^. In a proportion of patients with MASLD, simple steatosis can progress to NASH, characterized by hepatic inflammation, hepatocellular injury, and varying degrees of fibrosis with an increased risk of cirrhosis, liver failure, and hepatocellular carcinoma (HCC)^5,6^. The close relationship between insulin resistance and MASLD was demonstrated in studies using homeostatic model assessment (HOMA) as well in euglycemic–hyperinsulinemic clamps^7,8^. The metabolic comorbidities and cardiovascular morbidity and mortality are other important concerns in patients with MASLD^9–11^

The primary management strategy for MASLD involves lifestyle interventions focused on weight loss, dietary modifications and increased physical activity. The therapeutic options for MASLD are limited. Although lifestyle modifications and weight reduction are beneficial, they are often difficult to sustain^12^. In contrast, bariatric surgery and first-generation anti-obesity medications can be beneficial but less well tolerated^13^. The pharmacological therapies that have been used for the treatment of MASLD includes insulin sensitizers (e.g., pioglitazone, metformin), lipid-lowering agents (e.g., statins, ezetimibe), antioxidants (e.g., vitamin E), and agents targeting liver fat metabolism and inflammation (e.g., elafibranor, obeticholic acid) with limited benefits^14^. The incretin mimetics GLP-1RA like liraglutide and semaglutide with proved glycemic control, weight reduction potential and cardiovascular benefits also appears promising in targeting MASLD^15^.

With the rising prevalence of MASLD globally and the limited effective treatment options, there is a significant unmet need for therapies targeting MASLD^12^. Several drugs targeting different pathophysiolog mechanisms are being explored. The clinical trial development programs are also in progress for GLP1 agonists, TRβ agonists, and pan-PPAR agonists with promising preliminary reports^16^. Direct drug-induced modulation of adipose tissue function and hepatic lipid metabolism (PPAR agonists, FGF21 analogues, THRβ agonists, lipogenesis inhibitors) have also demonstrated improvements in the various histological MASLD components, independent of changes in body weight^13^.

Among the drugs targeting hepatic lipid metabolism, Resmetirom a thyroid hormone receptor beta (THR-β) agonist, has been extensively studied for treatment of MASLD. THR-β activity is vital for the hepatic lipid metabolism. Resmetirom, also known as MGL-3196, is an orally administered liver directed, selective thyroid hormone receptor beta (THR-β) agonist that has shown promise in the treatment of MASLD^8^. Resmetirom functions by specifically targeting THR-β, which is highly expressed in liver and involved in regulating lipid metabolism and inflammation in the liver. By selectively activating THR-β, Resmetirom modulates genes responsible for lipid metabolism, increases hepatic fat metabolism and reduces lipotoxicity, thus leading to reduced hepatic fat accumulation and inflammation^17^. This β selectivity also protects against the systemic adverse effects of excess thyroid hormone activity which are mainly mediated by thyroid hormone receptor alpha (THR- α). In recent years, several clinical trials have been conducted to assess the effectiveness and safety of Resmetirom in the treatment of MASLD^18^. The phase 2 and phase 3 trials on Resmetirom showed efficacy of the molecule in the treatment of MASLD by decreasing liver fat content and markers of inflammation and fibrosis^19,20^. However, it is crucial to determine the pooled effect of Resmetirom to better understand its efficacy and safety profile in the treatment of these conditions. Thus, this systematic review and meta-analysis aimed to explore the efficacy and safety of Resmetirom for the treatment of MASLD.

## Materials and methods

### Study registration

The systematic literature review was conducted as per the Preferred Reporting Items for Systematic Reviews and Meta-Analysis (PRISMA) reporting guidelines^21^. The protocol was registered with the International Prospective Register of Systematic Reviews PROSPERO (CRD42024534453).

### Databases and search strategy

PubMed, Scopus, Web of Science, Cochrane library and Embase were searched for literature on Resmetirom for the treatment of MASLD. The studies were screened from 2014 to 2024 and restricted to English language. Additionally, Google Scholar, Clinical Trials (ClinicalTrials.gov), abstracts from meetings and references listed in the qualified research were screened to identify any missing papers. The search term used for database screening are ‘Resmetirom’ AND ‘metabolic dysfunction-associated steatotic liver disease’ OR ‘MASLD’ OR ‘non-alcoholic fatty liver disease’ OR ‘NAFLD’ OR ‘non-alcoholic steatohepatitis’ OR ‘NASH’.

### Study selection

The studies were selected based on the following inclusion criteria (i) Studies enrolling patients with biopsy confirmed NAFLD/NASH (ii) Age > 18 years, (iii) Randomised controlled trials (iv) Resmetirom intervention in drug naïve subjects (v) Placebo controlled trials (vi) studies reporting changes in hepatic fat content and adverse events/efficacy and safety parameters. The exclusion criteria included (i) studies with no placebo arm (ii) studies with other comparators (iii) studies with switch over designs (iv) studies with duration of exposure to Resmetirom of less than 3 months (v) Studies including patients with cirrhosis, decompensated liver disease, alcoholic liver disease and other causes of chronic liver diseases vi) Observational and prospective studies.

### Intervention

The experimental group were administered Resmetirom, while the control group received placebo. The meta-analysis was performed based on the Resmetirom dosage and analysis time points.

### Outcome measures

Outcome measures were efficacy and safety indicators. The primary outcome was change in Magnetic resonance imaging-proton density fat fraction (MRI-PDFF), a sensitive measure of hepatic fat. The secondary outcome measures included efficacy indicators such as changes in Low-density lipoprotein cholesterol (LDL-C), triglycerides, lipoprotein (a) (Lpa), Apolipoprotein-B, Apolipoprotein- C III, Adiponectin, Reverse T3 and Cytokeratin(CK-18), and liver enzymes ALT (alanine transaminase), AST (aspartate aminotransferase), GGT (gamma-glutamyl transferase), thyroid function parameters such as FT3, FT4, thyroid stimulating hormone (TSH), thyroid-binding globulin (TBG), glycemic indicators such as fasting blood sugar (FBS), glycated hemoglubin (HbA1c) and fasting insulin, gonadal function parameters such as estradiol, testosterone, follicle stimulating hormone (FSH), luteinising hormone (LH) and sex hormone binding globulin (SHBG). The secondary outcome measures also included safety indicators such as treatment emergent adverse events, drug related serious adverse events, commonly reported adverse effects like diarrhoea and vomiting and Grade 3 changes in ALT.

### Data extraction

The primary literature search from the articles obtained from screening of the databases was performed by two researchers, who went through the titles and abstracts, and eliminated non-clinical trials and studies that did not include Resmetirom treatment and MASLD. The rescreening of the literature involved doing a preliminary screening of the entire body of work using the inclusion and exclusion criteria to determine the eligibility of RCTs. If two researchers disagreed on the study selection, the decision was taken after discussing with the third researcher. The following parameters were extracted from the eligible studies: first author, publication year, country, number of sites, study design, sample size, randomization, treatment duration, intervention, study criteria, outcome measures, baseline data such as age, sex, body mass index (BMI), comorbidities, dose, duration of treatment and outcome measures.

### Risk of *bias* and quality appraisal

The Cochrane Risk of Bias Tools were used to objectively evaluate the included RCTs. Three assessments are provided for the potential sources of bias risk resulting from inappropriate experimental procedures or the sample’s limitations during the study process: high risk, low risk and unclear risk. The software Revman 5.4 was utilized to produce the risk of bias summary. Two researchers carried out the tasks separately and disagreement in the decision were discussed with the third researcher.

### Statistical analysis

RevMan 5.4 software was used for the meta-analysis of the data. The effect indicators for the forest plot for continuous variables were expressed as the mean difference (MD) and 95% confidence interval (95% CI) and the odds ratio (OR)/risk ratio (RR) and 95% CI for dichotomous variables. I2 statistic were used to evaluate the statistical heterogeneity between studies. The sub-group analysis was performed based on intervention dosage and time points.

### Research involving human participants and/or animals

This study does not involve any human participants or animal performed by any of the authors.

## Results

**Figure 1:**
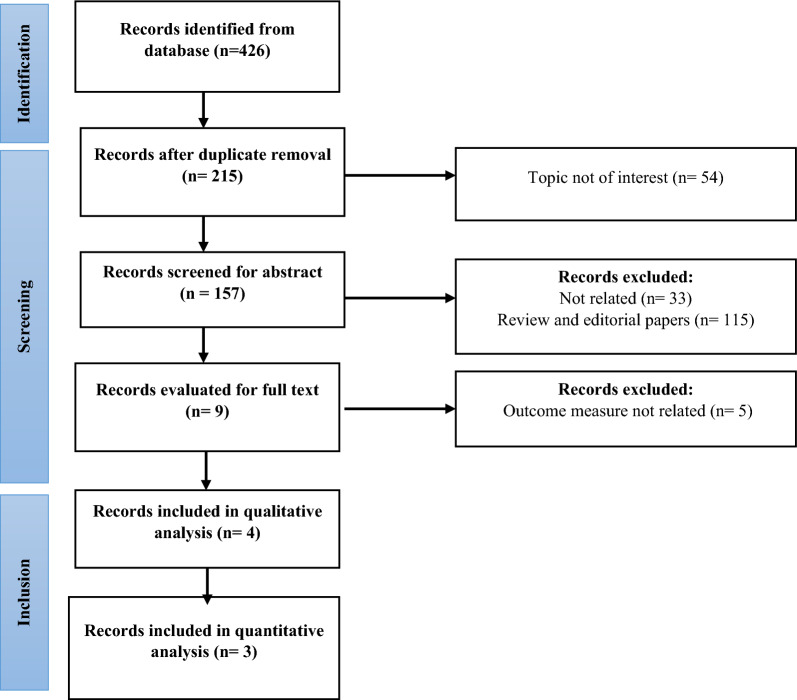
Flowchart illustrating the strategy used for screening studies in the database.

A total of 426 related titles were identified from literature search in this study, of which a total of 422 inappropriate titles/ abstracts were excluded. After the primary screening, 4 records were obtained for qualitative synthesis and 3 trials were included for meta-analysis. A total of 2265 subjects were included in the meta-analysis of whom 1569 received Resmetirom and 696 received placebo. The literature search strategy is shown in detail in the PRISMA flow chart (Fig. 1).

### Study characteristics

The studies MGL 3196 Phase 2 trial^18^, OLE trial^22^, MAESTRO-NAFLD-1^23^ and MAESTRO-NASH trial^24^, which investigated at efficacy and safety of Resmetirom in NAFLD were included. The baseline characteristics of all the studies included are depicted in Table 1. These studies were randomised placebo-controlled trials out of which three trials MGL 3196 Phase 2 trial, MAESTRO-NAFLD-1 and MAESTRO-NASH trial were double blind^18,23,24^ and OLE trial^22^ was an open label extension trial of MGL-3196^18^. Three trials met the criteria for the meta-analysis of the 80 mg Resmetirom dose compared with the placebo, while two trials examined the 100 mg dose.

**Table 1:** Baseline characteristic of included studies.

Study	MGL 3196 Phase 2: NASH	OLE MGL 3196 Phase 2: NASH	Phase 3: MAESTRO-NAFLD-1	Phase 3: MAESTRO- NASH
First Author	Stephen A. Harrison	Stephen A. Harrison	Stephen A. Harrison	Stephen A. Harrison
Year	2019	2021	2023	2024
Country	United States	United States	United States	United States
Sites	25	12	80	245
Study design	A multicentric, randomized, double-blind, Placebo- controlled, phase 2 trial	An active treatment open-label extension (OLE) study of randomized, double-blind, placebo-controlled phase 2 trial	A multicentric, Randomized, double-blind, placebo-controlled phase 3 trial	A multicentric, double-blind, randomized, placebo-controlled Phase 3 trial
Randomisation	A computer-generated simple randomisation schedule prepared by study administrators was used to randomly assign patients (2:1) to Resmetirom 80 mg or matching placebo administered orally once a day	Patients were randomized 2:1 to receive Resmetirom 80 mg or matching placebo, orally once a day	A stratified randomisation and web response system was used to assign three Double blind arms (100 mg Resmetirom, 80 mg Resmetirom or placebo) and one open-label (OL) (100 mg Resmetirom) arm	Randomization was performed with the use of an interactive Web response system. Patients were randomly assigned in a 1:1:1 ratio to receive Resmetirom at a dose of 80 mg or 100 mg or placebo, administered orally once daily
Sample size	125	31	1143	966
Groups	Resmetirom 80 mg (n = 84), and placebo (n = 41)	Resmetirom/Resmetirom (n = 17) and Placebo/ Resmetirom (n = 14)	100 mg Resmetirom (n = 325), 80 mg Resmetirom (n = 327), placebo (n = 320) and open-label (OL) 100 mg Resmetirom (n = 171)	Resmetirom at a dose of 80 mg (n = 322), Resmetirom- 100 mg (n = 323) or placebo (n = 321)
Treatment duration	36	36	52	52
Intervention	All patients randomly assigned to Resmetirom received an 80 mg dose for the first 4 weeks. At week 4, the Resmetirom dose was adjusted by 20 mg up or down or remained at 80 mg on the basis of the week 2 estimated AUC. Of 79 patients who completed at least week 4 and had Resmetirom exposure determined at week 2, 37 (47%) had an estimated total exposure to Resmetirom and had a dose reduction to 60 mg at week 4. The remaining 42 (53%) remained on 80 mg	Res/Res patients entering the OLE study initially continued on the dose of Resmetirom that they were on at the end of the main study. Pbo/Res patients were started on an 80-mg dose of Resmetirom on day 1 of the OLE study. Based on a trough and 4-h post-dose pharmacokinetic assessment at week 2, patients remained on the initial dose or were down-titrated or up-titrated by 20 mg at week 4, as determined by an unblinded reviewer. After the main study was unblinded, all patients in the OLE study had doses increased to at least 80 mg or 100 mg of Resmetirom	For all patients randomized to resmetirom treatment, dose adjustments could be triggered by an unblinded monitor. At week 12, the resmetirom dose was reduced by 20 mg if FT4 levels decreased from baseline by ≥ 30% (to < 0.7 ng dl − 1), the dose was further decreased to 60 mg at week 24. After week 24, no further resmetirom dose adjustments were permitted	A total of 1050 patients underwent randomization; 966 patients who had a fibrosis were randomly assigned to receive 80 mg of Resmetirom (n = 322), 100 mg of Resmetirom (n = 323), or placebo (n = 321)
Study criteria	Patients were eligible for screening if they were at least 18 years of age, had a diagnosis suggestive of NASH based on the presence of metabolic syndrome, elastography consistent with liver fibrosis and steatosis, or metabolic syndrome plus a previous liver biopsy consistent with NASH with non-cirrhotic fibrosis Patients were required to have at least 10% hepatic fat content on screening MRI-PDFF. Patients were excluded if they had a history of clinically significant alcohol consumption or use of drugs associated with NAFLD, hypothyroidism, uncontrolled type 2 diabetes, or a requirement for GLP-1, Statins	Patients were eligible for screening if they were at least 18 years of age, had a diagnosis suggestive of NASH based on the presence of metabolic syndrome, elastography consistent with liver fibrosis and steatosis, or metabolic syndrome plus a previous liver biopsy consistent with NASH with non-cirrhotic fibrosis. Patients were required to have at least 10% hepatic fat content on screening MRI-PDFF. Patients were excluded if they had a history of clinically significant alcohol consumption or use of drugs associated with NAFLD, hypothyroidism, uncontrolled type 2 diabetes, or a requirement for GLP-1, Statins	Male and female adults ≥ 18 years of age, suspected or confirmed diagnosis of NASH/NAFLD, must be on stable, standard care dyslipidemia therapy for ≥ 30 d before randomization, female patients with negative serum pregnancy test	Eligible patients were 18 years of age or older, histologic evidence of NASH and an NAFLD activity score of 4 or more, at least 50% of the total enrollment was required to have a fibrosis stage of F3. No more than 15% of the total enrollment could have a fibrosis stage of F1, primarily F1B, and no more than 3% of the total enrollment could have a fibrosis stage of F1A or F1C. Key exclusion criteria were alcohol consumption of more than 20 g per day for women and more than 30 g per day for men, a glycated hemoglobin level of more than 9.0% at screening, and causes of chronic liver disease other than noncirrhotic NASH
Outcome measures	Primary outcome: MRI-PDFF Secondary outcome: LDL-C, APO-B, APO-C, ALT, AST, GGT, Adiponectin, lipoprotein a, triglycerides, FBS, HbA1c, fasting insulin, FT3, FT4, TSH, TBG, Estradiol, testosterone, FSH, LH, SHBG and Adverse events	Primary outcome: MRI-PDFF. Secondary outcome: LDL-C, APO-B, APO-C, ALT, AST, GGT, Adiponectin, lipoprotein a, triglycerides, Adverse events	Primary outcome: MRI-PDFF. Secondary outcome: LDL-C, APO-B, APO-C, ALT, AST, GGT, Adiponectin, lipoprotein a, triglycerides, FT3, FT4, TSH, TBG, Estradiol, testosterone, FSH, LH, SHBG and Adverse events	Primary outcome: MRI-PDFF Secondary outcome: LDL-C, APO-B, APO-C, ALT, AST, GGT, Adiponectin, lipoprotein a, triglycerides, FBS, HbA1c, fasting insulin, FT3, FT4, TSH, TBG, Estradiol, testosterone, FSH, LH, SHBG and Adverse events
Age in years (mean ± sd)	Resmetirom (80 mg)—51·8 ± 10·4 Placebo- 47·3 ± 11·7	Res/Res—53.1 ± 11.8 Pbo/Res—42.4 ± 10.5	Resmetirom (100 mg)—55.9 ± 11.7 Resmetirom (80 mg)—56.2 ± 11.7 Placebo—55.7 ± 12.1	Resmetirom (100 mg)—57.0 ± 10.8 Resmetirom (80 mg)—55.9 ± 11.5 Placebo—57.1 ± 10.5
Male (%)	Resmetirom—38 (45) Placebo—24 (59)	Res/Res—8 (47.1) Pbo/Res—8 (57.1)	Resmetirom (100 mg)- 147 (45.4) Resmetirom (80 mg)–145 (44.3) Placebo—150 (47.2)	Resmetirom (100 mg) – 141 (43.7) Resmetirom (80 mg)—140 (43.5) Placebo—143 (44.5)
BMI, kg/m^2^ (mean ± sd)	Resmetirom—35·8 ± 6·2 Placebo—33·6 ± 5·8	Res/Res—34.5 ± 5.2 Pbo/Res—35.1 ± 5.2	Resmetirom (100 mg)–35.4 ± 6.4 Resmetirom (80 mg)–35.3 ± 5.9 Placebo—35.2 ± 5.8	Resmetirom (100 mg)- 36.2 ± 7.4 Resmetirom (80 mg)—35.5 ± 6.4 Placebo—35.3 ± 6.5
T2DM, n(%)	Resmetirom—36 (43%) Placebo—13 (32%)	Res/Res—9 (52.9) Pbo/Res—5 (35.7)	Resmetirom (100 mg)- 156 (48.1) Resmetirom (80 mg)—160 (48.9) Placebo—159 (50.0)	Resmetirom (100 mg)- 213 (65.9) Resmetirom (80 mg)—224 (69.6) Placebo—210 (65.4)
Hypertension, n (%)	Resmetirom—45 (53·6) Placebo—18 (43·9)	Res/Res—10 (58.8) Pbo/Res—6 (42.9)	Resmetirom (100 mg)- 246 (75.9) Resmetirom (80 mg)—249 (76.1) Placebo—242 (76.1)	Resmetirom (100 mg)—254 (78.6) Resmetirom (80 mg)—243 (75.5) Placebo—257 (80.1)
Dyslipidemia, n (%)	NR	NR	Resmetirom (100 mg)- 283 (87.3) Resmetirom (80 mg)—288 (88.1) Placebo—281 (88.4)	Resmetirom (100 mg)-236 (73.1) Resmetirom (80 mg)—229 (71.1) Placebo—224 (69.8)
Hypothyroidism, n (%)	NR	NR	Resmetirom (100 mg)- 34 (10.5) Resmetirom (80 mg)—39 (11.9) Placebo—35 (11.0)	Resmetirom (100 mg)—46 (14.2) Resmetirom (80 mg)—39 (12.1) Placebo—45 (14.0)
MRI-PDFF- %(sd)	Resmetirom (80 mg)—20·2% (6·8) Placebo- 19·6% (8·2)	Res/Res—21.0 (6.4) Pbo/Res—17.4 (7.6)	Resmetirom (100 mg)—18.1 (7.3) Resmetirom (80 mg)—17.7 (6.7) Placebo—17.8 (6.9)	Resmetirom (100 mg)—17.2(6.6) Resmetirom (80 mg)—18.2 (6.8) Placebo—17.8 (6.8)
LDL-c mg/dL (mean ± sd)	Resmetirom (80 mg) – 111.3 ± 30·4 Placebo-116·9 ± 30·0	Res/Res—53.1 ± 11.8 Pbo/Res—42.4 ± 10.5	Resmetirom (100 mg)—55.9 ± 11.7 Resmetirom (80 mg)—56.2 ± 11.7 Placebo—55.7 ± 12.1	Resmetirom (100 mg)—57.0 ± 10.8 Resmetirom (80 mg)—55.9 ± 11.5 Placebo—57.1 ± 10.5
Triglycerides mg/dL (mean ± sd)	Resmetirom (80 mg) – 161.1 ± 75.2 Placebo- 178.5 ± 82.4	Res/Res – 178.4 ± 72.0 Pbo/Res – 176.1 ± 110.1	Resmetirom (100 mg) – 174.1 ± 93.5 Resmetirom (80 mg) – 177.6 ± 94.4 Placebo – 186.8 ± 119.2	Resmetirom (100 mg)—188.7 ± 153.8 Resmetirom (80 mg)—189.2 ± 112.5 Placebo—184.1 ± 125.8
LP(a) nmol/L (mean ± sd)	Resmetirom (80 mg)—29·1 ± 44·7 Placebo- 36·9 ± 50·0	Res/Res—NR Pbo/Res—NR	Resmetirom (100 mg)—57.6 ± 77.6 Resmetirom (80 mg)—60.8 ± 77.5 Placebo—49.0 ± 70.2	Resmetirom (100 mg)—43.8 ± 60.8 Resmetirom (80 mg)—44.7 ± 61.1 Placebo—42.2 ± 62.7
APO-B mg/dL (mean ± sd)	Resmetirom (80 mg)—103·5 ± 22.8 Placebo- 104·1 ± 21.7	Res/Res—112 ± 30 Pbo/Res—110 ± 29	Resmetirom (100 mg)—95.5 ± 20.5 Resmetirom (80 mg)—98.1 ± 26.3 Placebo—95.1 ± 27.1	Resmetirom (100 mg)—95.9 ± 27.8 Resmetirom (80 mg)—98.4 ± 27.8 Placebo—97.8 ± 32.0
APO-CIII mg/dL (mean ± sd)	Resmetirom (80 mg)—10·6 ± 3.8 Placebo- 9·80 ± 3·7	Res/Res—11.2 ± 3.8 Pbo/Res—10.3 ± 3.3	Resmetirom (100 mg)—NR Resmetirom (80 mg)—NR Placebo—NR	Resmetirom (100 mg)—NR Resmetirom (80 mg)—NR Placebo—NR
ALT IU/L (mean ± sd)	Resmetirom (80 mg)—50·0 ± 29.2 Placebo- 60.1 ± 32.2	Res/Res – 58.5 ± 35.6 Pbo/Res – 70.6 ± 51.7	Resmetirom (100 mg) – 36.2 ± 25.2 Resmetirom (80 mg) – 37.1 ± 23.9 Placebo – 37.9 ± 30.4	Resmetirom (100 mg) – 56.3 ± 34.0 Resmetirom (80 mg) – 52.8 ± 27.3 Placebo – 54.7 ± 34.8
AST IU/L (mean ± sd)	Resmetirom (80 mg) – 38.0 ± 20.7 Placebo- 35.1 ± 17·7	Res/Res – 40.9 ± 24.8 Pbo/Res – 43.8 ± 16.4	Resmetirom (100 mg)—24.9 ± 12.4 Resmetirom (80 mg) – 25.3 ± 13.3 Placebo – 26.4 ± 16.4	Resmetirom (100 mg) – 42.5 ± 25.2 Resmetirom (80 mg) –38.2 ± 19.3 Placebo—40.7 ± 24.6
GGT IU/L (mean ± sd)	Resmetirom (80 mg) – 68.1 ± 60.7 Placebo- 48.5 ± 31.0	Res/Res – 76.6 ± 75.1 Pbo/Res – 57.6 ± 30.8	Resmetirom (100 mg) – 41.5 ± 31.8 Resmetirom (80 mg) – 46.1 ± 41.0 Placebo – 49.9 ± 62.1	Resmetirom (100 mg)—84.6 ± 99.0 Resmetirom (80 mg)- 84.3 ± 111.3 Placebo—75.7 ± 85.0

*NR* Not reported, *BMI* Body mass index, *MRI-PDFF* Magnetic resonance imaging proton density fat fraction, *LDL* Low-density lipoproteins, *LP(a)* Lipoprotein(a), *APO-B* Apolipoprotein B, *APO-CIII* Apolipoprotein C-III, *ALT* Alanine transaminase, *AST* Aspartate aminotransferase, *GGT* Gamma-glutamyl transferase, *FBS* Fasting blood sugar, *HbA1c* Glycated haemoglobin, *FT3* Free triiodothyronine, *FT4* Thyroxine, *TSH* Thyroid stimulating hormone, *TBG* Thyroxine-binding globulin, *FSH* Follicle-stimulating hormone, *LH* Luteinizing hormone, *SHBG* Sex hormone binding globulin.

## Study intervention

The intervention arm in all the studies received Resmetirom and the control arm received placebo. In MGL 3196 Phase 2 study^18^, drug was administered for 36 weeks without interruption. During the first four weeks of treatment with Resmetirom, patient was given an 80 mg dosage. Based on exposure measurements taken at week two, the 24-h Resmetirom area under the curve was calculated for each patient receiving Resmetirom. Following a measurement of their exposure to Resmetirom at week 2, 37 (47%) of the 79 patients had an estimated total exposure of more than 5500 ng*h/mL to Resmetirom plus an inactive metabolite at week 4, at which point their dose was lowered to 60 mg. The remaining 42 (53%) were maintained on 80 mg or, if their total exposure was 3000 ng*h/ml or less, had their dose increased to 100 mg.

Two main groups participated in the OLE trial^22^, an open label extension trial of MGL-3196^18^: former Resmetirom patients (Res/Res), many of whom were treated with a higher dose of Resmetirom during the OLE, and former placebo patients (Pbo/Res), who were treated with Resmetirom during the OLE study. Enrollment in the OLE study began in week 38 of the primary trial and continued for up to two months following the 2-week follow-up period without the study medication. Patients with Res/Res who were participating in the OLE study was taking Resmetirom at the completion of the main trial. Resmetirom 80 mg was administered to Pbo/Res patients on the first day of the OLE study. Based on post-dose pharmacokinetic evaluation at week 2, the increase in the dose of 20 mg at week 4 was determined. All participants in the OLE study received dosages of Resmetirom increased to at least 80 mg or 100 mg after the main study was unblinded.

In the MAESTRO-NAFLD-1 study 972 individuals were randomized to one of three DB arms^23^ (100 mg Resmetirom, n = 325, 80 mg Resmetirom, n = 327, or placebo, n = 320), and 171 patients were randomized to the open-label (OL) 100 mg Resmetirom arm. Two patients were randomly assigned to the placebo arm and one patient to the DB 100 mg Resmetirom arm, even though they did not receive the trial drug. The mean number of weeks of study drug exposure was 47 weeks in the OL Resmetirom arm (100 mg), 45 weeks in the DB Resmetirom arm (100 mg), 43 weeks in the DB Resmetirom arm (80 mg) and 45 weeks in the placebo arm. Adjustments in study doses were not frequent; 12 (2.4%) had their Resmetirom dose reduced from 100 to 80 mg and 2 (0.6%) had their dose decreased from 80 to 60 mg.

In MAESTRO-NASH study 1050 patients^24^ underwent randomization; 966 patients who had a fibrosis stage of F1B, F2, or F3 at baseline were randomly assigned to receive 80 mg of Resmetirom (322 patients), 100 mg of Resmetirom (323 patients), or placebo (321 patients). A total of 11 of 966 patients had a delay in their week 52 biopsy for reasons related to coronavirus disease 2019.

Since the study included multiple arms examining the effects of Resmetirom in MAESTRO-NAFLD and MAESTRO-NASH, a subgroup analysis was performed to ascertain the specific efficacy of Resmetirom at doses of 80 mg and 100 mg.

The MGL 3196 OLE phase 2 study^22^ was not included in the meta-analysis due to its distinct study design. In this study, subjects who were initially on Resmetirom were separated into treatment and placebo groups, which might potentially cause divergence in the pooled effect of the Resmetirom in the meta-analysis.

## Meta-analysis

### Efficacy of Resmetirom

**Figure 2:**
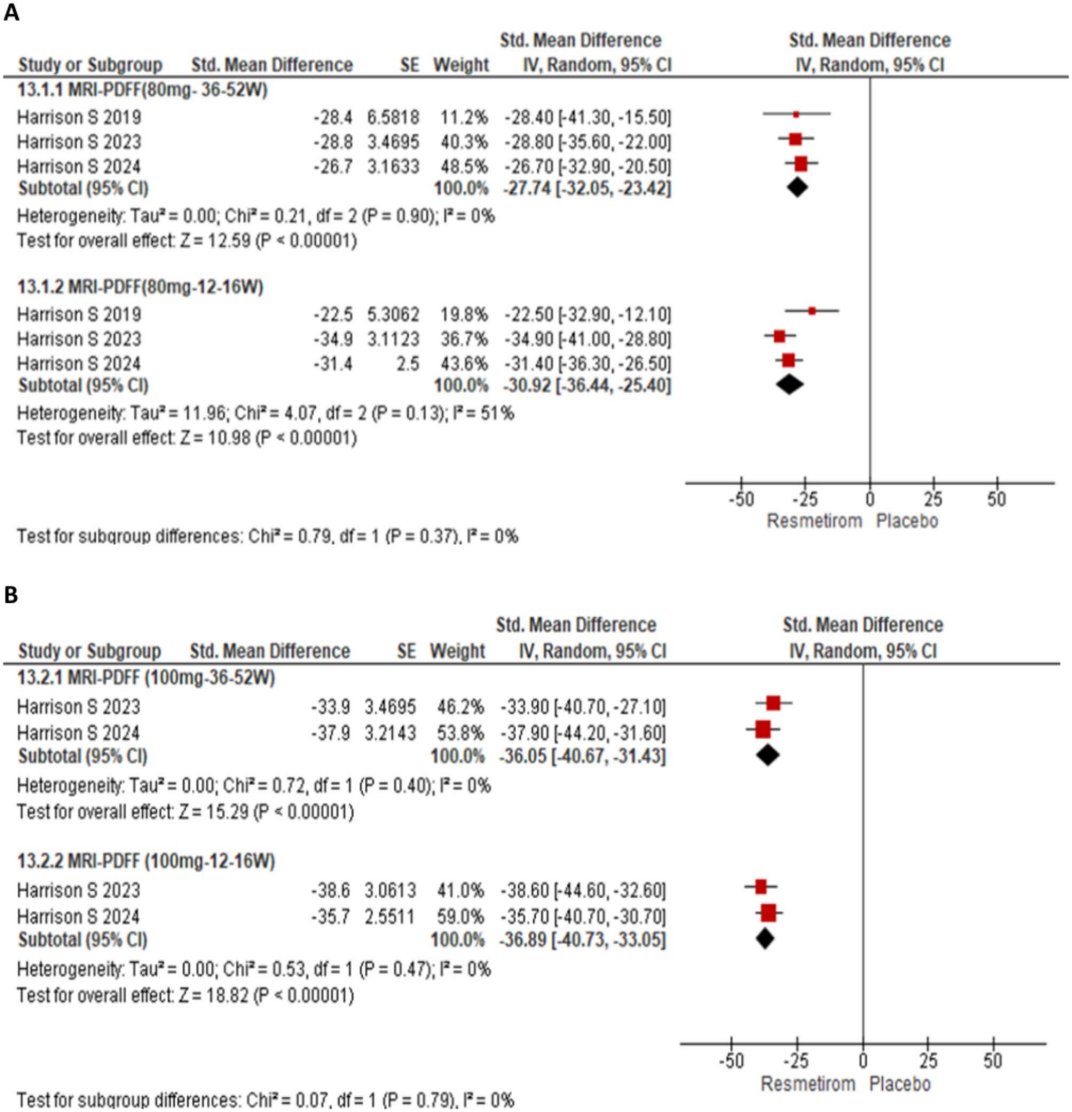
Forest plot showing change in MRI-proton density fat fraction from baseline to week 36–52 and to 12–16 week at dose of (**A**) 80 mg Resmetirom and (**B**) 100 mg Resmetirom compared to placebo group.

The meta-analysis of the efficacy of Resmetirom included total 2234 subjects (1552 in Resmetirom and 685 in placebo). The meta-analysis of change in MRI-PDFF for three trials at dose of 80 mg Resmetirom involved 655 subjects in Resmetirom arm and 627 in placebo arm and the dose of 100 mg included 591 subjects in Resmetirom and 589 in placebo arms. The forest plot was examined to assess the change in MRI-PDFF for Resmetirom at a dosage of 80 mg at 36–52-week period and also at weeks 12–16 for three trials. Similarly, the same time-point analysis was conducted for Resmetirom at a dosage of 100 mg for two trials (Fig. 2). A random effects model was applied for this analysis and the pooling of data from these studies showed a significant reduction in MRI-PDFF with 80 mg Resmetirom as compared to the placebo [SMD − 27.74% (95% CI − 32.05 to − 32.42), p < 0.00001] at 36–52 weeks as well as at 12–16 weeks [SMD − 30.92% (95% CI − 36.44 to − 25.40), p < 0.00001] (Fig. 2A). Similarly, a significant decrease was noted in MRI-PDFF with Resmetirom 100 mg dose at week 36–52 [SMD − 36.05% (95% CI − 40.67 to − 31.43), p < 0.00001] and week 12–16 [SMD − 36.89% (95% CI − 40.73 to − 33.05), p < 0.00001] (Fig. 2B).

The meta-analysis of three trials looking at changes in LDL-C showed a significant reduction in LDL-C in the Resmetirom 80 mg arm compared to placebo at 12–24 week [SMD − 12.43 mg/dL (95% CI − 15.16 to − 9.71), p < 0.00001] and at 36–52 week [SMD − 12.68 mg/dL (95% CI − 16.64 to − 8.73), p < 0.00001] (figure S1A). Similar effect was seen for LDL-C at Resmetirom 100 mg dose at 12–24 weeks [SMD − 14.61 mg/dL (95% CI − 18.32 to − 10.89), p < 0.00001] and 36–52 weeks [SMD − 15.63 mg/dL (95% CI − 22.49 to − 8.78), p < 0.00001] (figure S2B). The forest plot for triglycerides showed a significant reduction in triglycerides in the 80 mg Resmetirom arm compared to the placebo at 24 weeks [SMD − 18.09 mg/dL (95% CI − 24.30 to − 11.89), p < 0.00001] and 36–52 weeks [SMD − 20.73 mg/dL (95% CI − 27.13 to − 14.33), p < 0.00001] (figure S2A). Similar effect of reduction in triglyceride was seen for with Resmetirom 100 mg dose at 24 weeks [SMD − 19.67 mg/dL (95% CI − 26.18 to -13.17), p < 0.00001] and 36–52 week [SMD − 23.73 mg/dL (95% CI − 29.85 to − 17.61), p < 0.00001] (Figure S2B).

The meta-analysis for Lipoprotein (a) revealed a significant reduction with 80 mg Resmetirom group compared to placebo group at 12–24 weeks [SMD − 21.52 nmol/L (95% CI − 37.40 to -5.65), p = 0.008] and 36–52 weeks [SMD − 26.12 nmol/L (95% CI − 35.42 to -16.83), p < 0.00001] (figure S3A). Likewise, significant reduction in Lipoprotein (a) was found with Resmetirom 100 mg at 12–24 weeks [SMD − 31–87 nmol/L (95% CI − 38.52 to − 25.21), p < 0.00001] and 36–52 weeks [SMD − 30.37 nmol/L (95% CI − 36.82 to − 23.91), p < 0.00001] (figure S3B). The meta-analysis revealed statistically significant reductions in Apolipoprotein B levels in the 80 mg Resmetirom group compared to the placebo group at 24 weeks [SMD − 16.62 mg/dL (95% CI − 18.85 to -14.38), p < 0.00001] and 36–52 weeks [SMD − 18.16 mg/dL (95% CI − 23.81 to − 12.52), p < 0.00001] (figure S4A). Similarly, significant reductions in Apolipoprotein B were observed with the 100 mg Resmetirom at 24 weeks [SMD − 19.44 mg/dL (95% CI − 21.62 to -17.25), p < 0.00001] and weeks 36–52 [SMD − 20.41 mg/dL (95% CI − 25.69 to -15.14), p < 0.00001] (figure S4B). A significant difference was seen in Apolipoprotein—C III levels between the Resmetirom 80 mg and the placebo group at week 24 [SMD − 14.82 mg/dL (95% CI − 20.71 to -8.94), p < 0.00001] and weeks 36–52 [SMD − 23.06 mg/dL (95% CI − 32.47 to − 13.65), p < 0.00001] (figure S5A). Resmetirom 100 mg showed similar results at week 24 [SMD − 20.06 mg/dL (95% CI − 24.47 to − 15.65), p < 0.00001] and weeks 36–52 [SMD − 24.16 mg/dL (95% CI − 28.82 to − 19.51), p < 0.00001] (figure S5B).

Three studies that reported liver enzymes were included in the metanalysis examining the pooled effect of resmetirom on liver enzymes for Resmetirom doses at 80 mg and 100 mg. There was a significant reduction in alanine transaminase with 80 mg Resmetirom at 36–52 weeks [SMD − 16.73 IU/L (95% CI − 26.00 to − 7.45), p = 0.0004] and 100 mg Resmetirom at 36–52 weeks [SMD − 18.29 IU/L (95% CI − 33.45 to − 3.11), p = 0.002] (figure S6A). A significant reduction was observed in aspartate aminotransferase with 80 mg Resmetirom dose at 36–52 weeks [SMD − 11.29 IU/L (95% CI − 19.37 to − 3.21), p = 0.006], but no significant difference was seen with 100 mg Resmetirom at 36–52 weeks [SMD -14.56 IU/L (95% CI − 35.23 to 6.11), p = 0.17] (figure S6B). Similar results were observed for gamma-glutamyl transferase for 80 mg Resmetirom at 48 weeks [SMD -25.40 IU/L (95% CI − 45.14 to − 5.66), p = 0.01] which was significant, whereas for 100 mg Resmetirom at 48 weeks [SMD − 22.37 IU/L (95% CI − 47.07 to 2.32), p = 0.08] no significant reduction was found (figure S6C).

Meta-analysis was also performed for adiponectin, cytokeratin-18 (CK-18) and reverse T3 for 80 mg and 100 mg Resmetirom dose at 36–52 weeks (figure S7). The forest plot for adiponectin showed a significant improvement with Resmetirom 80 mg [SMD 0.90 mg/L (95% CI 0.63 to 1.17), p < 0.00001] and 100 mg [SMD 0.89 mg/L (95% CI 0.08 to 1.69), p = 0.03] at 36–52 weeks (figure S7A). There was a significant reduction in reverse T3 with 80 mg Resmetirom [SMD − 3.82 ng/dl (95% CI − 4.90 to − 2.75), p < 0.00001] and 100 mg Resmetirom [SMD − 4.58 ng/dl (95% CI − 5.81 to − 3.34), p < 0.00001] at 52 weeks (figure S7B). Similarly, significant reduction was seen in cytokeratin-18 level with Resmetirom 80 mg [SMD − 121.19 U/L (95% CI − 163.48 to − 78.91), p < 0.00001] and 100 mg [SMD -124.07 U/L (95% CI − 205.51 to − 42.63), p = 0.003] compared to placebo at 52 weeks (Figure S7C).

The metanalysis of the two studies that reported glycemic parameters showed that there were no significant differences in the FBS [SMD − 2.84 mg/L (95% CI − 6.36 to 0.67), p = 0.11], HbA1c [SMD 0.10% (95% CI − 0.22 to 0.43), p = 0.54] and fasting insulin [SMD − 0.26 mIU/L (95% CI − 7.93 to 7.41), p = 0.95] between the Resmetirom 80 mg and placebo group. However, only one study on 100 mg dose of Resmetirom reported a significant reduction in FBS [SMD − 5.60 mg/L (95% CI − 9.50 to − 1.70), p = 0.005] in the Resmetirom group, though HbA1c [SMD 0.05% (95% CI − 1.90 to 2.00), p = 0.96] and fasting insulin levels [SMD -1.80 mg/L (95% CI − 12.60 to 9.00), p = 0.74] did not show significant differences (Figure S8).

The meta-analysis of thyroid function tests of three studies revealed no significant changes in FT3 levels for both the 80 mg [SMD 0.04 ng/dL (95% CI − 0.01 to 0.09), p = 0.15] and 100 mg [SMD − 0.03 ng/dL (95% CI − 0.09 to 0.02), p = 0.25] Resmetirom doses (Figure S9A). However, there was a significant reduction in FT4 levels in the Resmetirom group compared to placebo for both 80 mg [SMD − 0.15 ng/dL (95% CI − 0.21 to − 0.10), p < 0.00001] and 100 mg dose [SMD − 0.24 ng/dL (95% CI − 0.26 to − 0.22), p < 0.00001] (Figure S9B). No significant changes were observed in TSH levels with the 80 mg [SMD -0.14 mIU/L (95% CI − 0.42 to 0.14), p = 0.32], and 100 mg Resmetirom dose [SMD 0.09 mIU/L (95% CI − 0.46 to 0.63), p = 0.75] compared to placebo (Figure S9C). Additionally, significant reductions in TBG levels were observed with both the 80 mg [SMD − 1.60 mg/L (95% CI − 2.23 to − 0.97), p < 0.00001] and 100 mg [SMD − 1.12 mg/L (95% CI − 2.68 to 0.45), p = 0.16] Resmetirom doses compared to placebo (Figure S9D).

All three trials reported effect on sex hormones in both women and men. The meta-analysis for estradiol in females showed a significantly higher levels with Resmetirom 100 mg [SMD 19.52 ng/L (95% CI 0.62 to 38.42), p = 0.04], but no changes were found for the Resmetirom 80 mg [SMD 11.99 ng/L (95% CI − 0.57 to 24.55), p = 0.06] (Figure S10A) as compared to placebo. Similar findings were also seen in males with a significantly higher estradiol levels with both the 80 mg [SMD 7.24 ng/L (95% CI 4.32 to 10.16), p < 0.00001] and 100 mg [SMD 10.90 ng/L (95% CI 8.69 to 13.11), p < 0.00001] Resmetirom doses as compared to placebo (Figure S10B). Testosterone levels showed a significant increase in Resmetirom group for both the 80 mg and 100 mg Resmetirom doses in females and males (Figure S10C and D). A significant higher level of SHBG was observed in Resmetirom group compared to placebo for both males and females (Figure S11A and S11B).

There were no significant changes in FSH levels for the 80 mg [SMD 0.13 IU/L (95% CI − 1.32 to 1.58), p = 0.86] and 100 mg doses [SMD 0.93 IU/L (95% CI − 1.41 to 3.27), p = 0.44] in females, as well as for the 100 mg dose [SMD 1.11 IU/L (95% CI − 0.06 to 2.29), p = 0.06] in males. However, a significant increase in FSH levels was observed in the Resmetirom group for the 80 mg [SMD 0.83 IU/L (95% CI 0.20 to 1.47), p = 0.01] dose (Figure S12A and B). A significant increase in LH levels was seen in Resmetirom group in males for both the 80 mg [SMD 0.50 IU/L (95% CI − 0.01 to 1.02), p = 0.05] and 100 mg [SMD 1.41 IU/L (95% CI 0.24 to 2.59), p = 0.02] Resmetirom doses, whereas no significant changes were observed in females for either dose (Figure S12C and D).

### Adverse events

**Figure 3:**
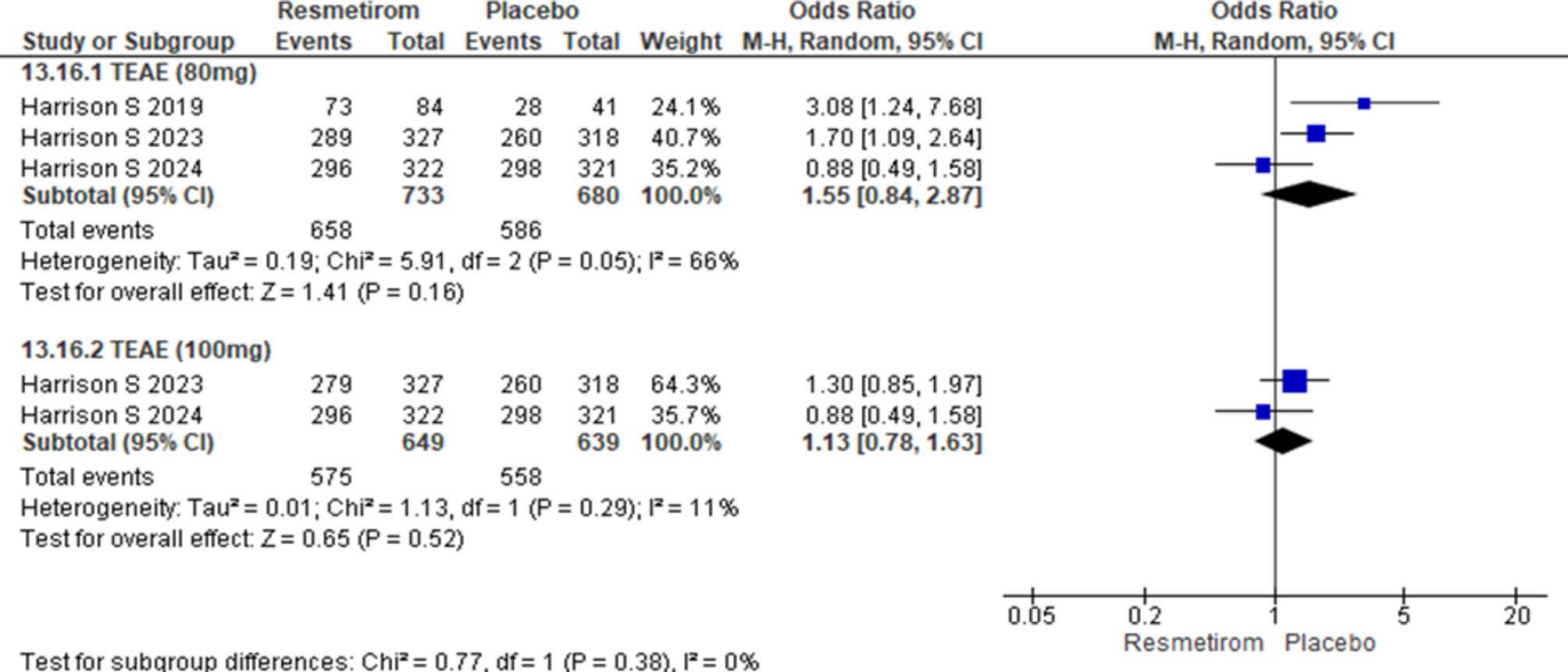
Forest plot showing Treatment emergent adverse events at Resmetirom dose of 80 mg and 100 mg *vs*. placebo group.

**Figure 4:**
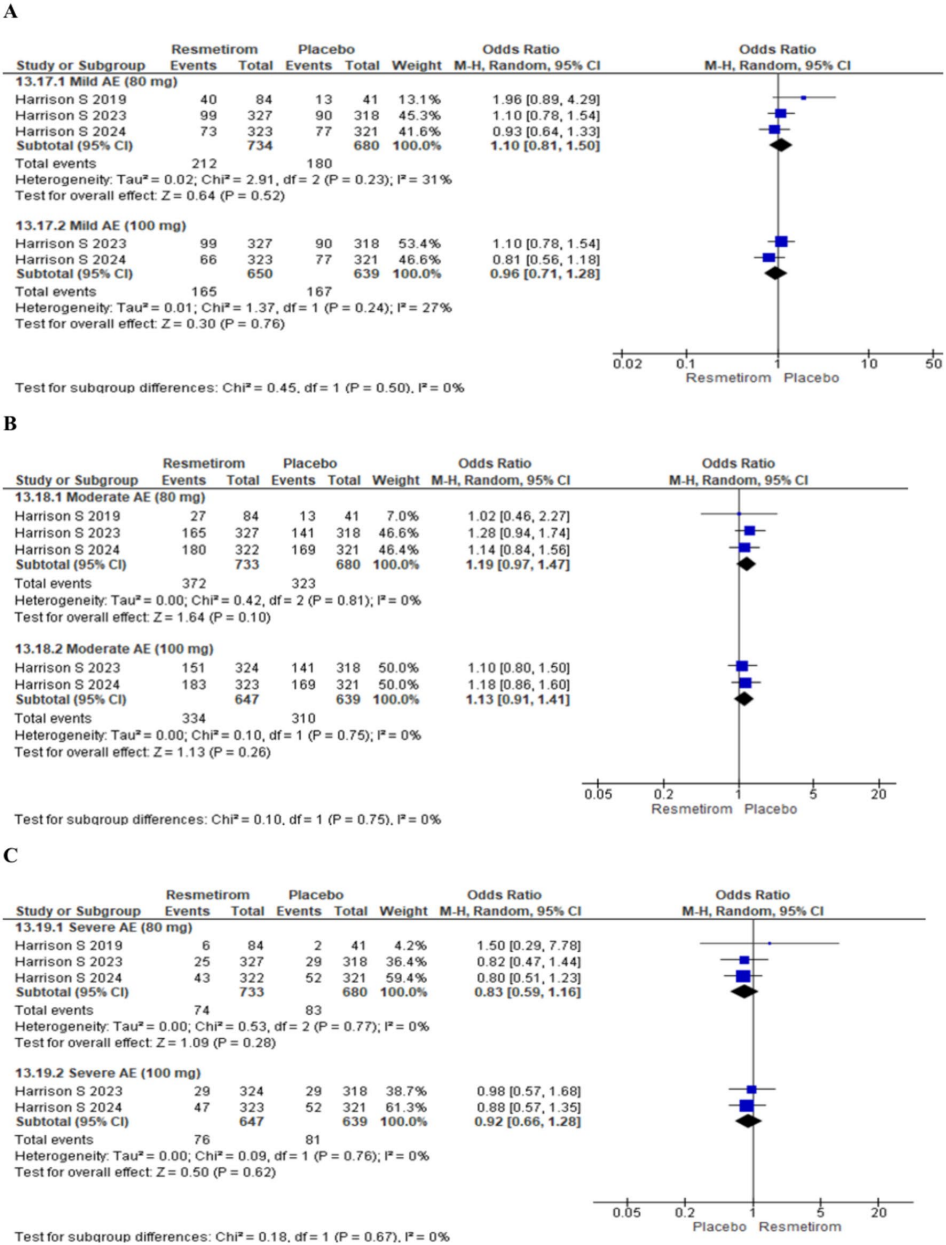
Forest plot for (**A**) mild adverse events, (**B**) moderate adverse events and (**C**) severe adverse events at Resmetirom dose of 80 mg and 100 mg *vs*. placebo group.

Two trials reported treatment emergent adverse events at 80 mg Resmetirom and two trials at 100 mg Resmetirom 100 mg. The forest plot for overall treatment emergent adverse events did not show any significant difference between Resmetirom and Placebo at Resmetirom dose of 80 mg [OR 1.55 (95% CI 0.84 to 2.87), p = 0.16] and 100 mg [OR 1.13 (95% CI 0.78 to 1.63), p = 0.52] (Fig. 3). Similarly, there was no significant difference between Resmetirom and placebo for mild adverse events at 80 mg Resmetirom [OR 1.10 (95% CI 0.81 to 1.50), p = 0.52] and 100 mg Resmetirom [OR 0.96 (95% CI 0.71 to 1.28), p = 0.76] (Fig. 4A), moderate adverse events at 80 mg [OR 1.19 (95% CI 0.97 to 1.47), p = 0.10] and 100 mg [OR 1.13 (95% CI 0.91 to 1.41), p = 0.26] (Fig. 4B) and severe adverse events at 80 mg [OR 0.83 (95% CI 0.59 to 1.16), p = 0.28] and 100 mg [OR 0.92 (95% CI 0.66 to 1.28), p = 0.62] (Fig. 4C).

**Figure 5:**
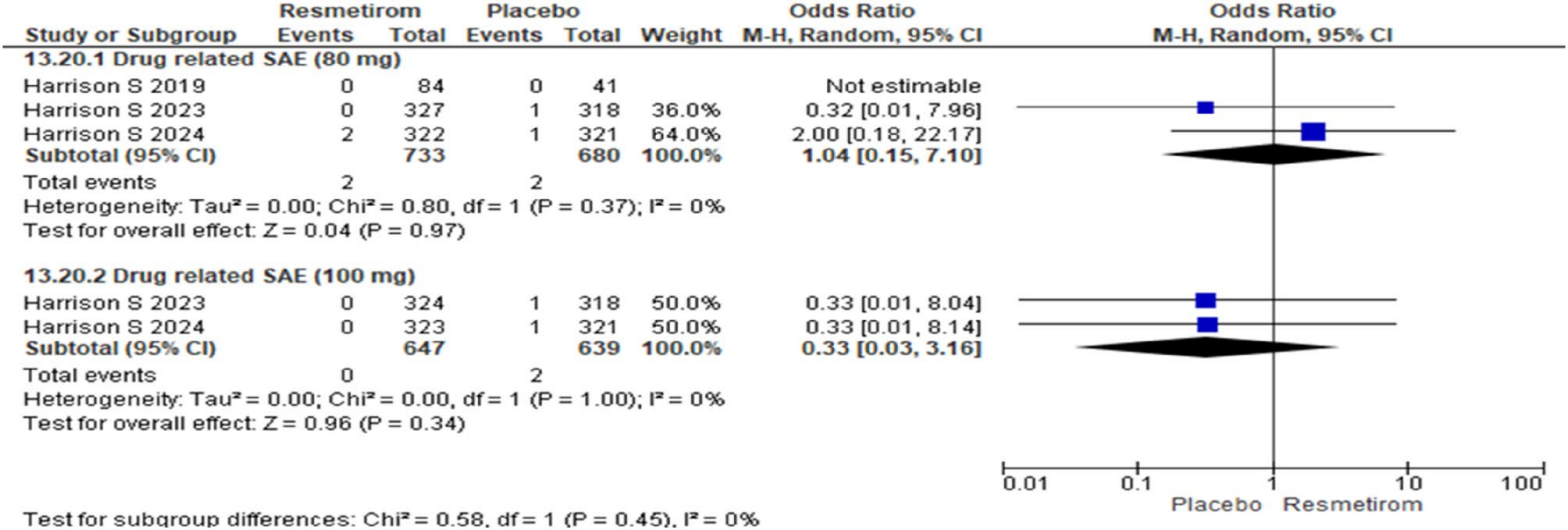
Forest plot depicting the drug related serious adverse event in Resmetirom at dose 80 mg and 100 mg.

**Figure 6:**
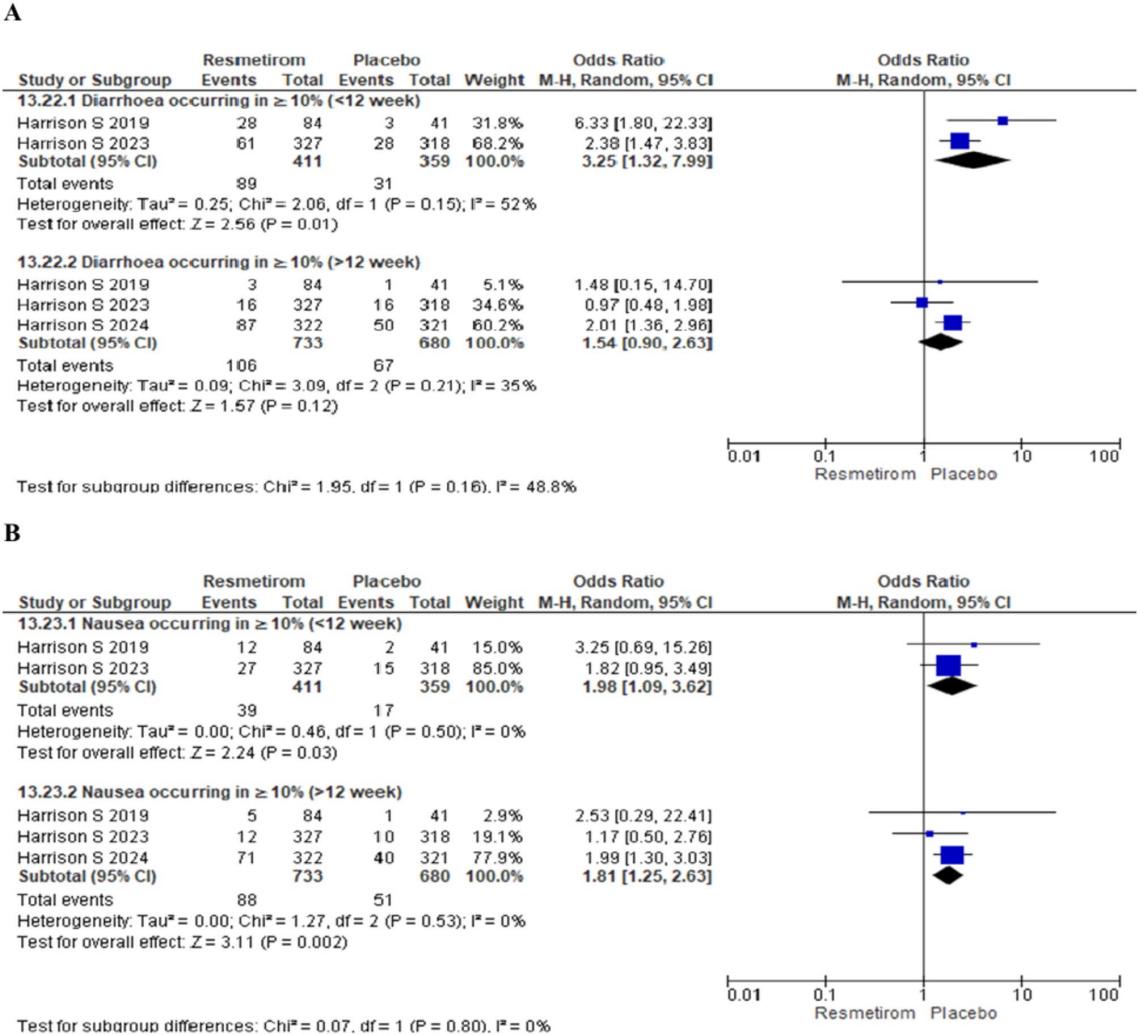
Forest plot for adverse event diarrhea and nausea occurring in ≥ 10% for < 12 week and > 12 week.

The forest plots for drug related serious adverse event in Resmetirom at dose 80 mg [OR 1.04 (95% CI 0.15 to 7.10), p = 0.97] and 100 mg [OR 0.33 (95% CI 0.03 to 3.16), p = 0.34] did not show any significant difference (Fig. 5). The meta-analysis for adverse event diarrhoea occurring in ≥ 10% was significantly higher with Resmetirom for < 12 weeks [OR 3.25 (95% CI 1.32 to 7.99), p = 0.01] whereas no significant difference was found between Resmetirom and placebo for duration of more > 12 weeks [OR 1.54 (95% CI 0.90 to 2.63), p = 0.12] (Fig. 6A). Adverse event nausea occurring in ≥ 10% was significantly higher in Resmetirom group in < 12 weeks mg [OR 1.98 (95% CI 1.09 to 3.62), p = 0.03]and > 12 weeks mg [OR 1.85 (95% CI 1.25 to 2.63), p = 0.002] (Fig. 6B).

The forest plot for grade 2 laboratory changes in ALT (defined as an increase of 1.5 to 3 times above the upper limit of normal laboratory range) showed a significant change in placebo group compared to Resmetirom group [OR 0.25 (95% CI 0.07 to 0.94), p = 0.04] (figure S13).

### Risk of bias

The four trials included in the study were rated as having a low risk of bias for random sequence generation, allocation concealment, blinding, incomplete outcome data and selective reporting as described in Figure S14.

## Discussion

Resmetirom is as an orally active, liver directed selective thyroid hormone receptor-β agonist, targeting key metabolic pathways involved in hepatic lipid metabolism and inflammation. It is highly selective with 28 times greater selectivity for THR-β versus THR-α than triiodothyronine. By modulating these pathways, it offers a targeted approach to managing MASLD^25^. This meta-analysis demonstrated a significantly better efficacy of resmetirom in improving liver fat fraction, atherogenic lipid profile, lipoproteins and liver enzymes and the safety profile.

Thyroid hormones acting through hepatic THR-β play an important role in lipid metabolism at the hepatic level by regulating the hepatic triglyceride storage and cholesterol metabolism^26^. Thyroid hormones also regulate the circulating cholesterol, triglycerides and lipoproteins. MASLD has been proposed to be a state of diminished liver thyroid hormone levels or hepatic hypothyroidism^27^. Reduction in THR-β activity in the liver is proposed to promote progression of MASLD. Resmetirom which is a liver directed selective thyroid hormone receptor-β agonist targets this pathophysiological abnormality. Resmetirom by re-establishing TRβ activity, decreases lipotoxicity and increases hepatic fat metabolism leading to improvement in MASLD^22,28^. The relative liver-specific expression of THR-β is essential for enhancing bile acid production, promoting fat oxidation, and reducing triglyceride and cholesterol levels^25^. Furthermore, because liver-specific organic anion carrying polypeptides mediate Resmetirom's uptake, which is liver-directed, hepatic specificity is ensured^29^. With a 28-fold selectivity for THR-β over THR-α, Resmetirom may be able to prevent the negative systemic consequences of excess thyroid hormone in the heart and bones, which are mostly mediated by THR-α^30^. Resmetirom does not appear to directly affect vascular smooth muscle or endothelial cells because its mechanism of action is mediated by THR-β, and its uptake is liver-directed. Nevertheless, it is thought to enhance mitochondrial biogenesis, mitophagy, liver-mitochondrial respiration rate, and mitochondrial oxidation, which reduces the risk of cardiovascular disease^31^.

Our metanalysis showed the efficacy of Resmetirom in improving hepatic fat content as demonstrated by significant reduction in MRI-PDFF with Resmetirom at 80 mg and 100 mg as compared to placebo. Hepatic fat content can be accurately quantified using MRI-PDFF, an imaging technique. Adults with NASH who achieved a ≥ 30% reduction in hepatic fat from baseline (measured by MRI-PDFF) had greater odds of achieving NASH reduction and resolution, according to a systematic review and meta-analysis by Stine et al., which suggests that this threshold could be used as a marker for improvement in NASH^32^.

In the MGL-3196-05 study, at 12 weeks and 36 weeks, the proportion of patients with a 30% or more relative fat reduction was higher in the Resmetirom group compared to the placebo group. Higher resmetirom exposure (AUC ≥ 2700 ng*h/mL) or higher SHBG response (change from baseline ≥ 75% at week 12 and 88% at week 36) were associated with greater absolute and relative reductions in hepatic fat from baseline at 12 weeks (− 8·5% [0·7]) and 36 weeks (–41·1% [4·8]), indicating that patients with higher plasma (drug exposure) and liver exposures were more effective in lowering hepatic fat. The treatment target of at least 30% fat reduction was likewise achieved by a higher percentage of patients in the high exposure group at 12 weeks and 36 weeks. Similar outcomes were seen in subgroups based on demographics, diabetes status, and liver fibrosis stage. According to a per-protocol study, individuals who continued on 80 mg or 100 mg after week 4 achieved 50·5% relative and 10·8% absolute fat loss at week 36 in MRI-PDFF, compared to those on 60 mg^18^.

As demonstrated in the MAESTRO-NASH trial, NASH resolution with no worsening of fibrosis was achieved in 25.9% of patients with 80 mg of resmetirom and 29.9% with 100 mg of resmetirom, compared with 9.7% of those who received a placebo. Additionally, there was a noticeable improvement in at least one stage of fibrosis^19^. By reducing liver stiffness (P = 0.015), N-terminal type III collagen pro-peptide (P = 0.0004), and PRO-C3/C3M (matrix metalloproteinase-degraded C3), a measure of net fibrosis production, Resmetirom also showed improvement in fibrosis^22^.

In this metanalysis, the lipid parameters LDL-c and triglyceride and the lipoproteins such as Lipoprotein(a), Apolipoprotein B and Apolipoprotein C3 showed a significant reduction in Resmetirom group compared to placebo suggesting the beneficial effects of Resmetirom on lipid profile and apolipoprotein. However, there was no significant impact on glycemic parameters. Resmetirom showed beneficial effect on liver with improvement in the liver enzymes such as ALT, AST and GGT. Further, the other biomarkers such as adiponectin, Reverse T3 and cytokeratin 18 (CK-18) also showed significant reduction with Resmetirom suggesting the efficacy of resmetirom in improving various components of MASLD. The potential positive cardiovascular benefits of these changes demand further evaluation in larger long-term studies.

The intrahepatic activity of thyroid hormones is dependant on serum thyroid hormone levels as well as the hepatic deiodinases. The switch from thyroid hormone-activating, Deiodinase 1(D1) to thyroid hormone-deactivating, Deiodinase 3(D3) enzyme is proposed to mediate the intrahepatic hypothyroidism in MASLD even in euthyroid state^33^. The lipotoxicity associated with MASLD leads to an enhanced conversion of T4 to the inactive metabolite rT3 and decreased conversion of prohormone T4 to the active hormone T3. A liver-targeted THR-β-selective agonist Resmetirom was developed to treat this underlying pathology in MASLD patients^34^. It has been previously shown that there is a slight decrease in FT4 with Resmetirom, which is believed to be caused by a decline in rT3 in the liver and an increase in T4 to T3 conversion^35^. Similar finding of reduced FT4 was also seen in this metanalysis. Significant sexual dimorphism is seen in MASLD, which is probably due to the significant influence of sex hormones on hepatic and extrahepatic lipid, carbohydrate, and protein metabolism^34^. In patients with MASLD, abnormalities of the sex hormone axes are particularly common. SHBG, a surrogate marker of hepatic exposure to resmetirom, is a downstream target of hepatic TRβ agonism^33^. Resmetirom treatment resulted in a substantial increase in SHBG levels, indicating TRβ activation. Further studies are required to understand the clinical impact of these changes in thyroid and sex steroid hormones. It is important to be aware of these alterations while interpreting sex steroids in patients on Resmetirom.

This metanalysis also looked at the safety of Resmetirom and found no major difference in the overall treatment emergent adverse events, mild AE, moderate AE and severe AE and in drug-related serious adverse events between Resmetirom and placebo. However, it was noted that the incidence of diarrhoea and nausea was found to be higher with Resmetirom therapy. Resmetirom’s unique mechanism of action suggests potential synergies with existing therapies and lifestyle interventions, to optimize treatment outcomes in MASLD patients. Resmetirom, with its promising clinical profile, presents an opportunity to address the unmet needs and improve outcomes for MASLD patients^21^. The introduction of resmetirom diversifies the therapeutic armamentarium for MASLD, providing clinicians with an additional tool to tailor treatment strategies based on individual patient characteristics and disease severity^18^. Beyond symptom management, resmetirom holds potential for disease modification by targeting underlying pathophysiological mechanisms implicated in MASLD progression. This could lead to long-term benefits such as reduced risk of advanced liver disease and related complications^25^.

The strength of this study is inclusion of good quality randomised controlled trails with low risk of bias and analysis of efficacy and adverse effects of Resmetirom and its impact of thyroid and gonadal function tests. The study's limitations include the small number of available trials with different study phases, which may potentially affect the accuracy of the combined efficacy results. Additionally, assessing the effectiveness of the Resmetirom across various stages of MASLD and histological characteristics was not feasible. Additional research will be required to determine long-term and off-target impacts. Further long-term studies examining the histological changes in the liver could confirm the beneficial effects of resmetirom in reducing inflammation and fibrosis and adverse hepatic complications in MASLD.

In conclusion, resmetirom represents a promising advancement in the management of MASLD, offering targeted therapy with the potential to improve liver parameters and address the unmet needs of the global burden of MASLD. While opportunities for targeted therapy and disease modification are evident, challenges such as long-term safety, patient adherence, and regulatory considerations must be addressed to understand its full potential in clinical practice.

### Supplementary Information

Supplementary Figures.

## Data Availability

Data is provided within the manuscript or supplementary information files.
