# Supplementary material for: Efficacy and safety of Resmetirom, a selective thyroid hormone receptor-β agonist, in the treatment of metabolic dysfunction-associated steatotic liver disease (MASLD): a systematic review and meta-analysis
Source: Sci Rep. 2024 Aug 26;14:19790. doi: 10.1038/s41598-024-70242-8 (PMC11347689; doi:10.1038/s41598-024-70242-8)
Supplement: Supplementary file 1 — Supplementary Figures. [file 41598_2024_70242_MOESM1_ESM.docx]

**Efficacy and Safety of Resmetirom, a selective thyroid hormone receptor-β agonist, in the treatment of Metabolic Dysfunction-associated Steatotic Liver Disease (MASLD): A Systematic Review and Meta-analysis**

Renuka Suvarna^1^, Sahana Shetty^1*^, Joseph M Pappachan^2^

| **A**  **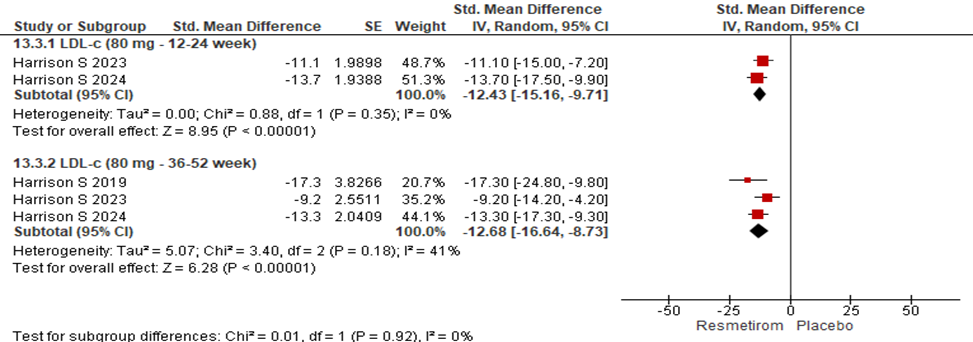** |
| --- |
| **B**  **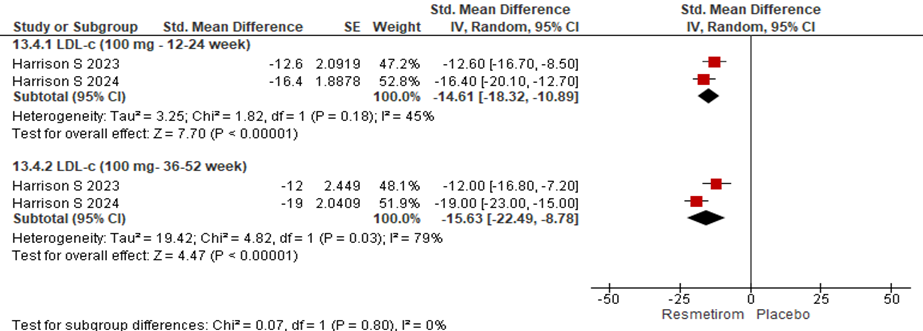** |

**Figure S1**. Forest plot showing changes in LDL-c for A) Resmetirom dose 80 mg at 12-24 week and 36-52-week, B) Resmetirom dose 100 mg at 12-24 week and 36-52 week.

| **A**  **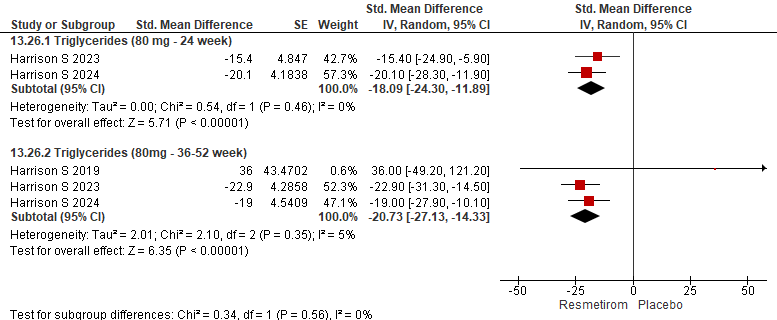** |
| --- |
| **B**  **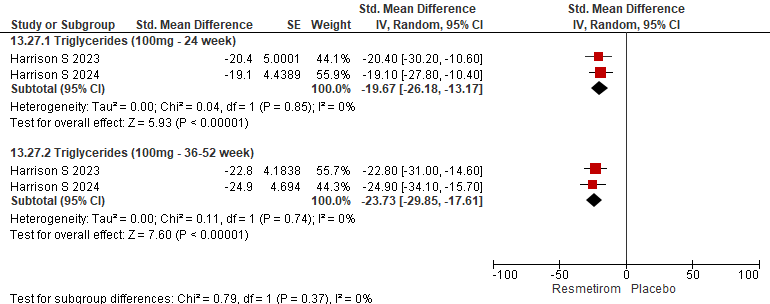** |

**Figure S2**. Forest plot showing changes in triglycerides for A) Resmetirom dose 80 mg at 12-24 week and 36-52-week, B) Resmetirom dose 100 mg at 12-24 week and 36-52 week.

| **A**  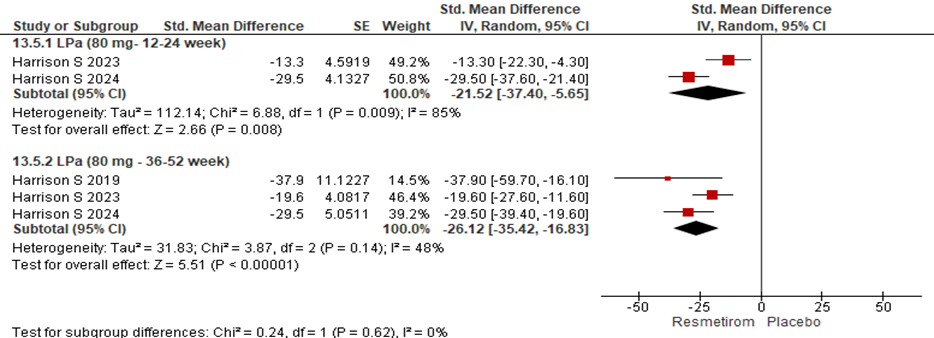 |
| --- |
| **B**  **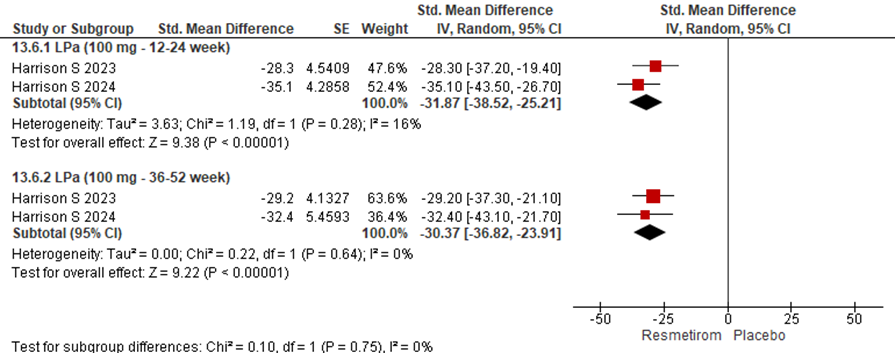** |

**Figure S3**. Forest plot showing changes in Lipoprotein(a) for A) Resmetirom dose 80 mg at 12-24 week and 36-52-week, B) Resmetirom dose 100 mg at 12-24 week and 36-52 week.

| **A**  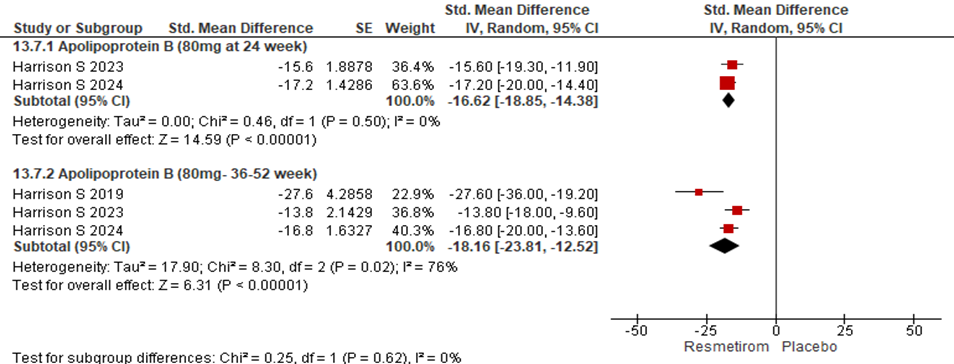 |
| --- |
| **B**  **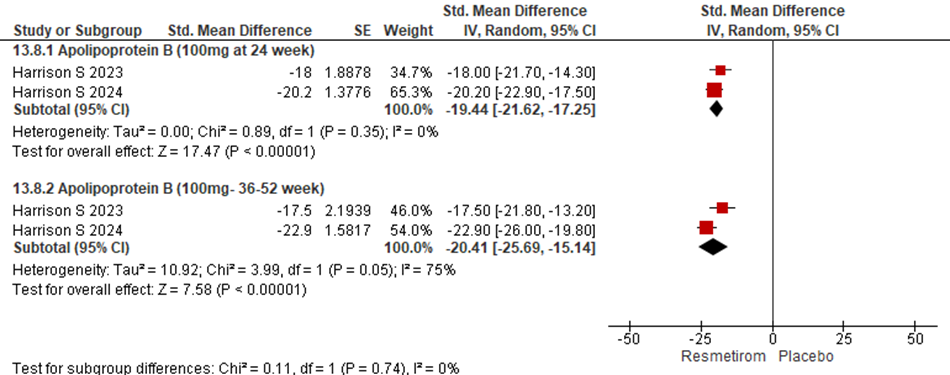** |

**Figure S4**. Forest plot showing changes in Apolipoprotein B for A) Resmetirom dose 80 mg at 24 week and 36-52-week, B) Resmetirom dose 100 mg at 24 week and 36-52 week.

| **A**  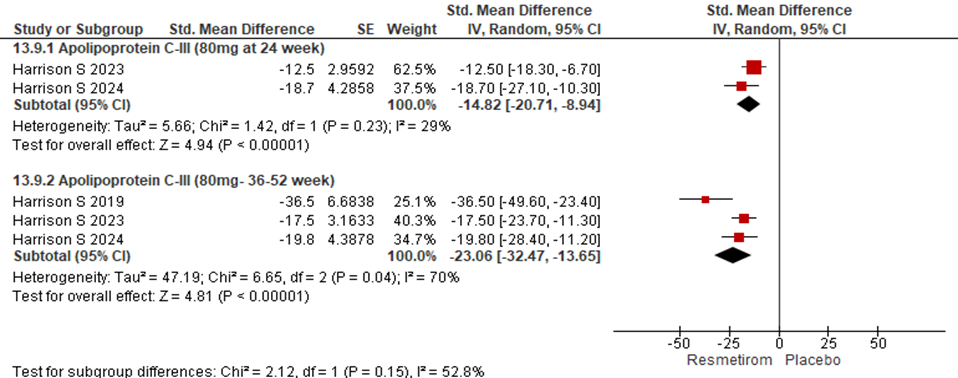 |
| --- |
| **B**  **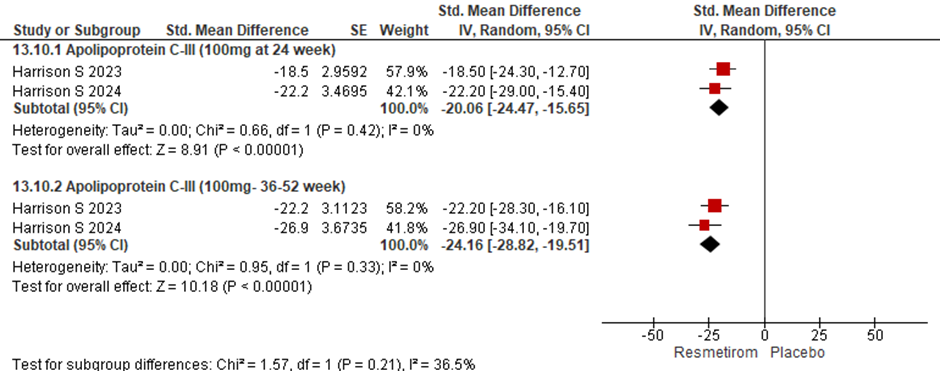** |

**Figure S5**. Forest plot showing changes in Apolipoprotein - C III for A) Resmetirom dose 80 mg at 24 week and 36-52-week, B) Resmetirom dose 100 mg at 24 week and 36-52 week.

| **A**  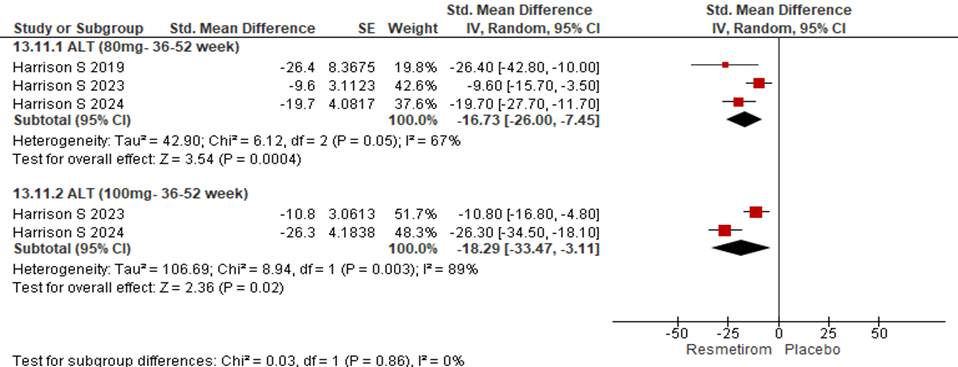 |
| --- |
| **B**  **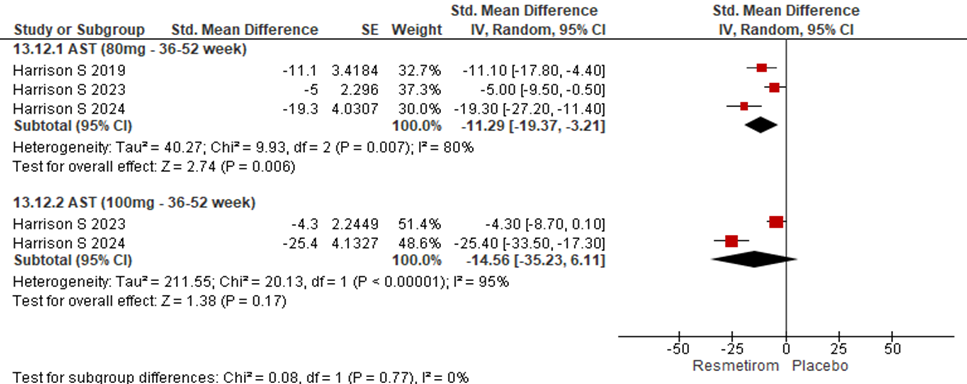** |
| **C**  **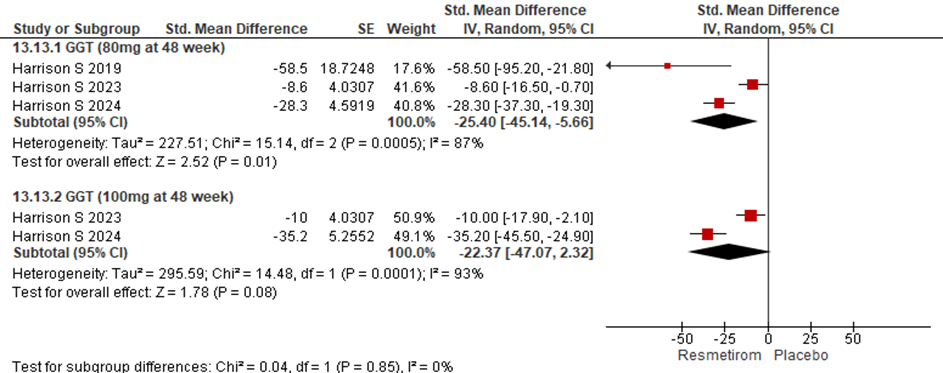** |

**Figure S6**. Forest plot showing changes in levels of liver enzymes A) ALT, B) AST and C) GGT at Resmetirom dose 80 mg and 100 mg.

| **A**  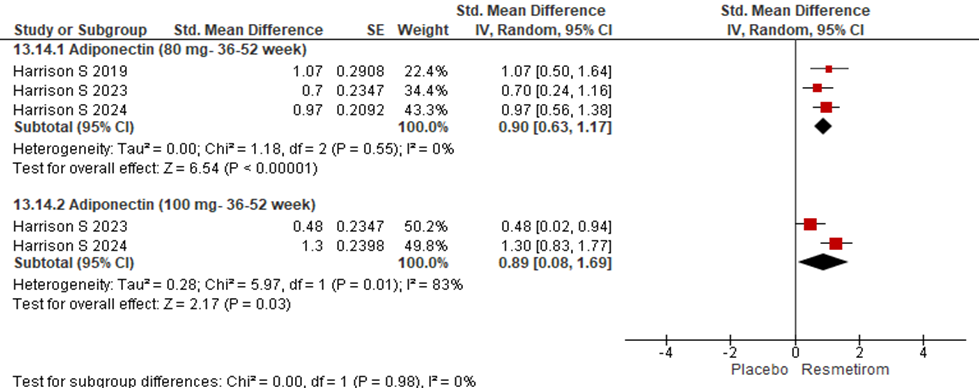 |
| --- |
| **B**  **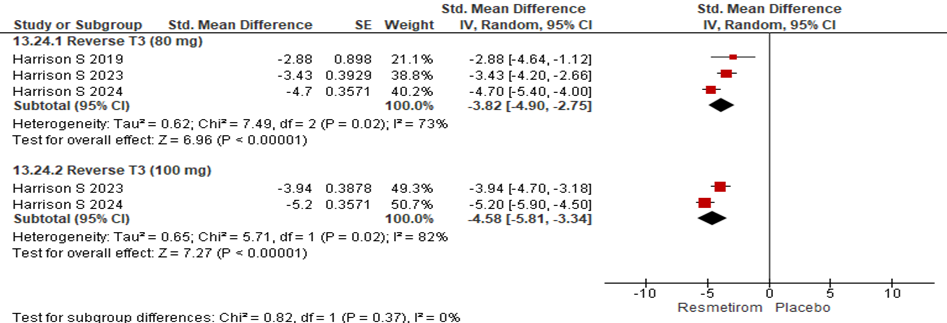** |
| **C**  **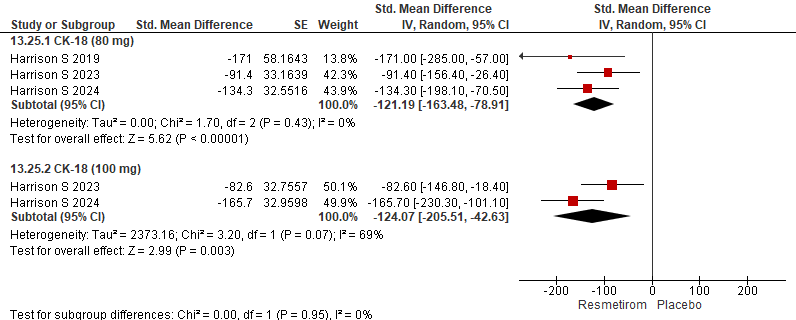** |

**Figure S7**. Forest plot showing changes in levels of A) Adiponectin, B) Reverse T3 and C) cytokeratin 18 (CK-18) at Resmetirom dose 80 mg and 100 mg.

| **A**  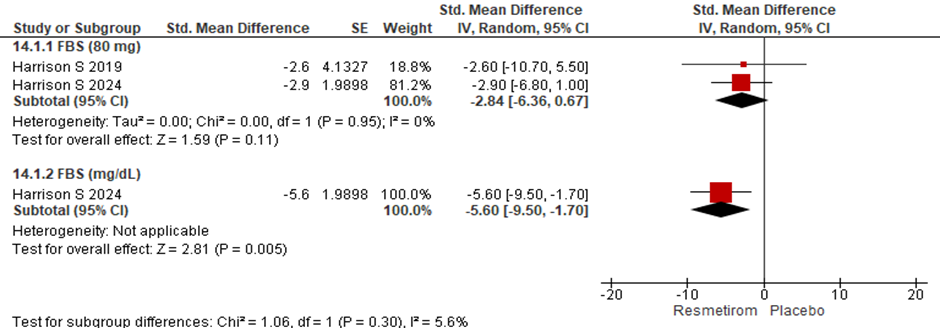 |
| --- |
| **B**  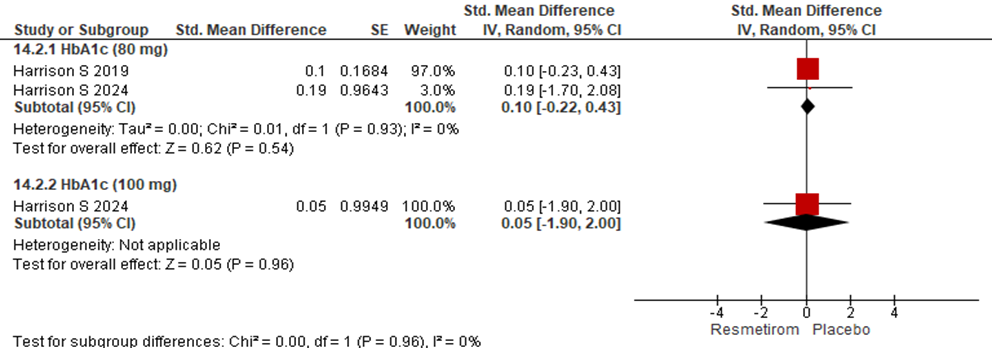 |
| **C**  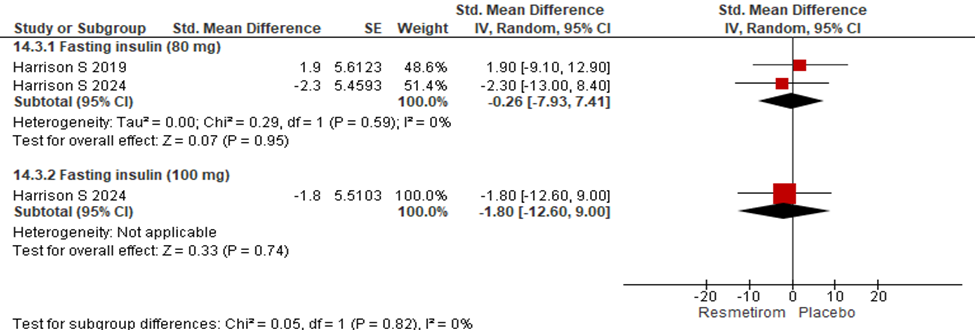 |

**Figure S8**. Forest plot showing changes in levels of A) FBS, B) HbA1c and C) fasting insulin at Resmetirom dose of 80 mg and 100 mg.

| **A**  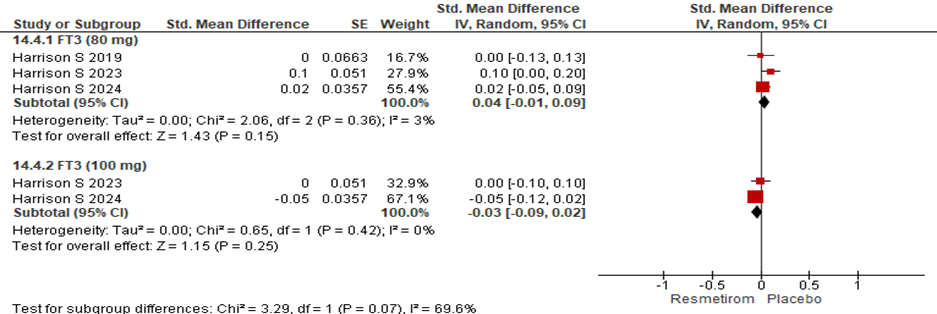 |
| --- |
| **B**  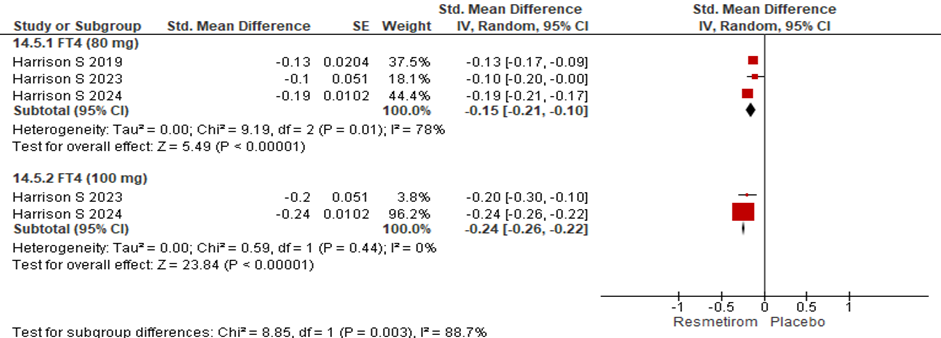 |
| **C**  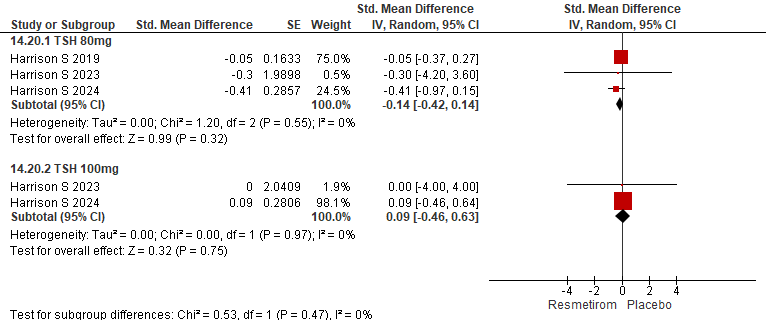 |
| **D**  **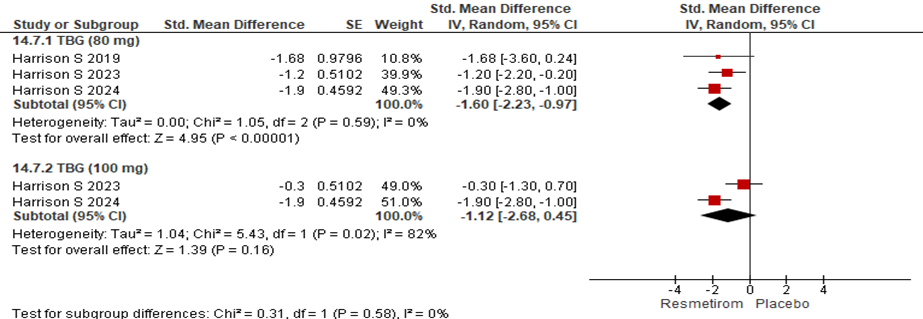** |

**Figure S9**. Forest plot showing changes in levels of thyroid function parameters A) FT3, B) FT4, C) TSH and D) TBG at Resmetirom dose of 80 mg and 100 mg.

| **A**  **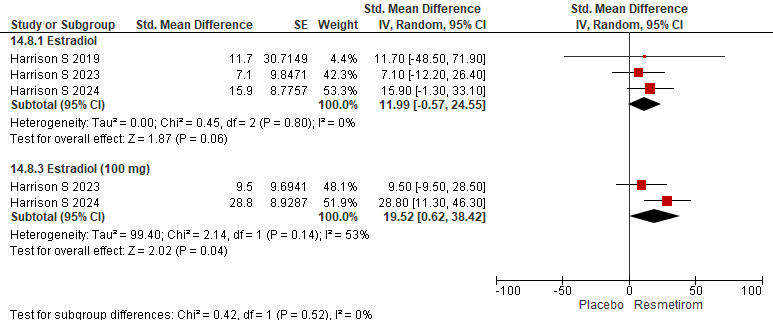** |
| --- |
| **B**  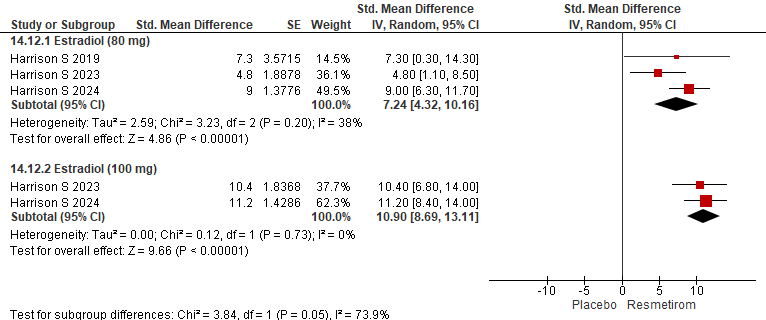 |
| **C**  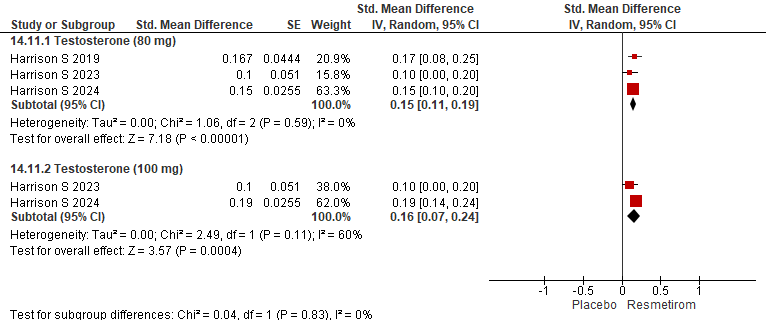 |
| **D**  **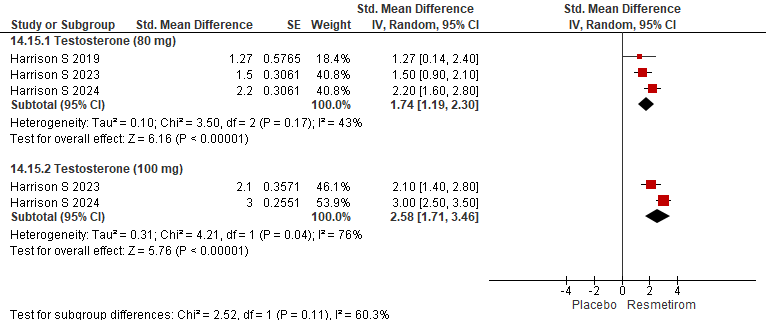** |

**Figure S10**. Forest plot showing changes in levels of sex hormone function parameters A) Estradiol in females, B) Estradiol in males, C) testosterone in females and D) testosterone in males at Resmetirom dose of 80 mg and 100 mg.

| **A**  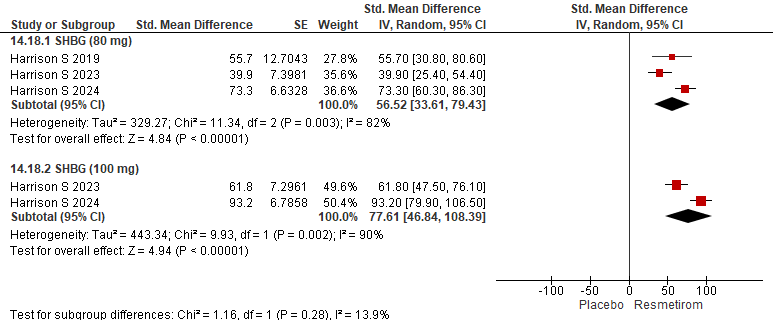 |
| --- |
| **B**  **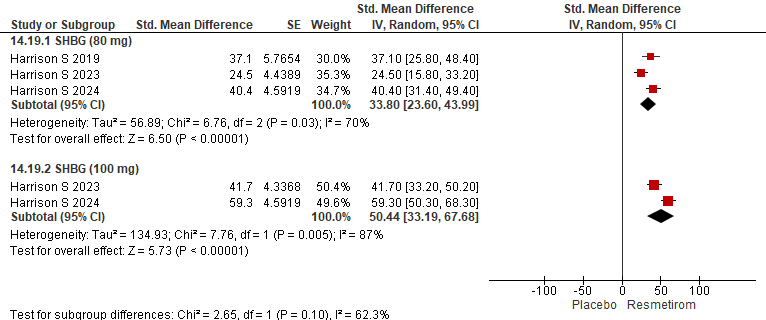** |

**Figure S11**. Forest plot showing changes in levels of A) SHBG in females and B) SHBG in males at Resmetirom dose of 80 mg and 100 mg.

| **A**  **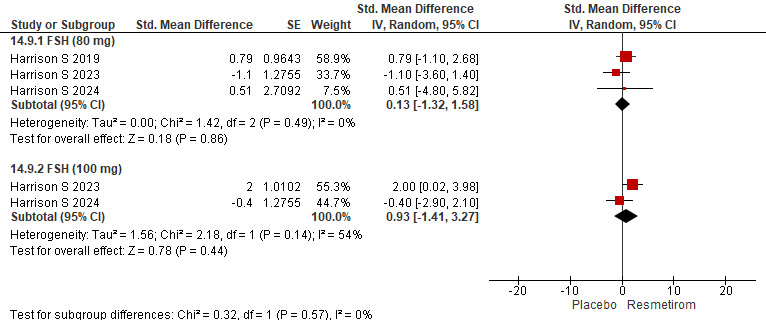** |
| --- |
| **B**  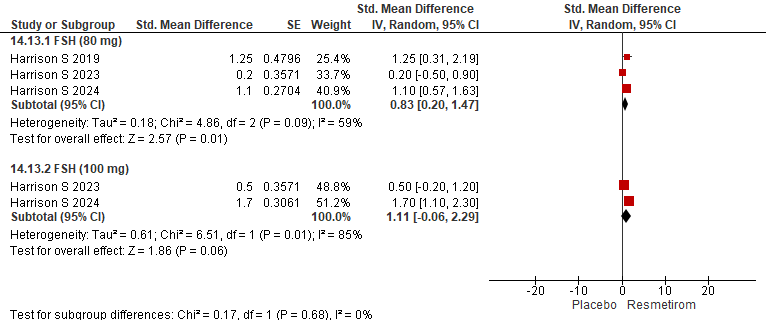 |
| **C**  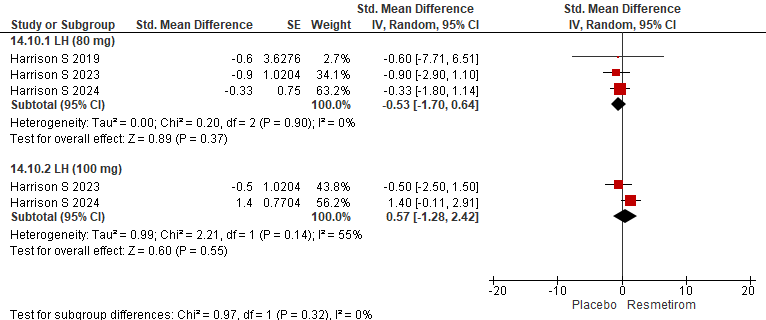 |
| **D**  **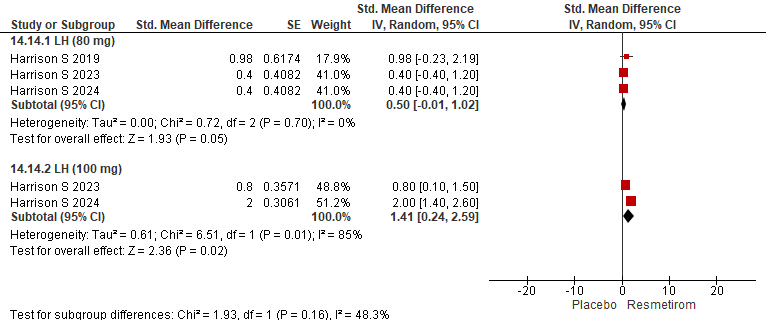** |

**Figure S12**. Forest plot showing changes in levels of gonadal function parameters A) FSH in females, B) FSH in males, C) LH in females and D) LH in males at Resmetirom dose of 80 mg and 100 mg.


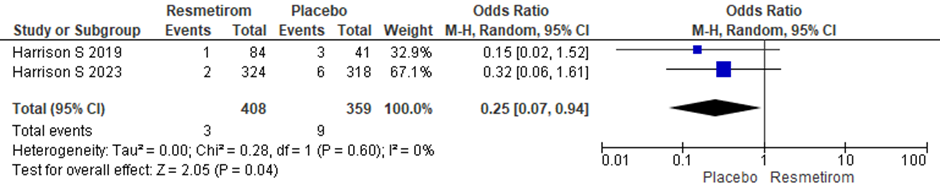


**Figure S13.** Forest plot depicting the grade 2 laboratory changes in ALT.

| **A**  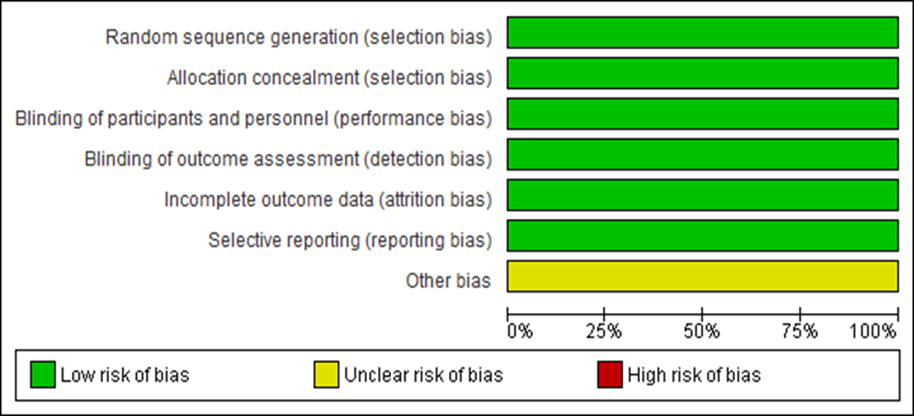 |
| --- |
| **B**  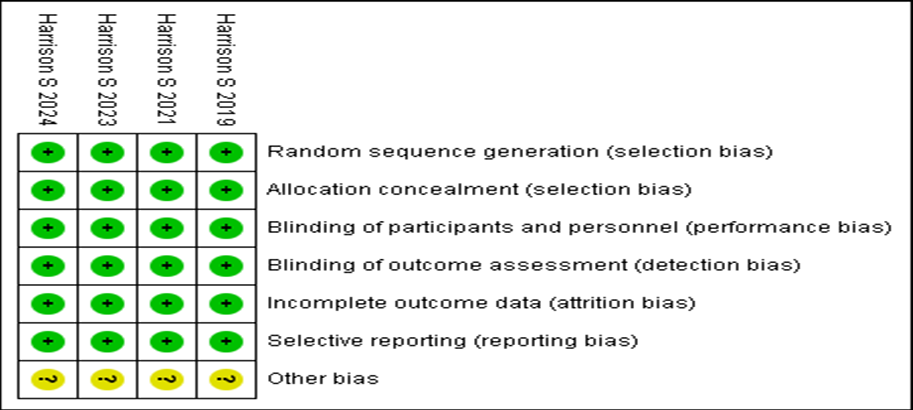 |

**Figure S14:** Risk of bias graph and summary of eligible study.
